# Population Differentiation and Demographic History of the *Cycas taiwaniana* Complex (Cycadaceae) Endemic to South China as Indicated by DNA Sequences and Microsatellite Markers

**DOI:** 10.3389/fgene.2019.01238

**Published:** 2019-12-23

**Authors:** Xin-Hui Wang, Jie Li, Li-Min Zhang, Zi-Wen He, Qi-Ming Mei, Xun Gong, Shu-Guang Jian

**Affiliations:** ^1^Guangdong Provincial Key Laboratory of Applied Botany, South China Botanical Garden, Chinese Academy of Sciences, Guangzhou, China; ^2^College of Resources and Environment, University of Chinese Academy of Sciences, Beijing, China; ^3^School of Life Sciences, Sun Yat-sen University, Guangzhou, China; ^4^Key Laboratory for Plant Diversity and Biogeography of East Asia, Kunming Institute of Botany, Chinese Academy of Sciences, Kunming, China

**Keywords:** *Cycas taiwaniana* complex, genetic diversity, phylogeographic structure, population dynamics, conservation

## Abstract

Historical geology, climatic oscillations, and seed dispersal capabilities are thought to influence the population dynamics and genetics of plants, especially for distribution-restricted and threatened species. Investigating the genetic resources within and among taxa is a prerequisite for conservation management. The *Cycas taiwaniana* complex consists of six endangered species that are endemic to South China. In this study, we investigated the relationship between phylogeographic history and the genetic structure of the *C. taiwaniana* complex. To estimate the phylogeographic history of the complex, we assessed the genetic structure and divergence time, and performed phylogenetic and demographic historical analyses. Two chloroplast DNA intergenic regions (cpDNA), two single-copy nuclear genes (SCNGs), and six microsatellite loci (SSR) were sequenced for 18 populations. The SCNG data indicated a high genetic diversity within populations, a low genetic diversity among populations, and significant genetic differentiation among populations. Significant phylogeographical structure was detected. Structure and phylogenetic analyses both revealed that the 18 populations of the *C. taiwaniana* complex have two main lineages, which were estimated to diverge in the Middle Pleistocene. We propose that *Cycas fairylakea* was incorporated into *Cycas szechuanensis* and that the other populations, which are mainly located on Hainan Island, merged into one lineage. Bayesian skyline plot analyses revealed that the *C. taiwaniana* complex experienced a recent decline, suggesting that the complex probably experienced a bottleneck event. We infer that the genetic structure of the *C. taiwaniana* complex has been affected by Pleistocene climate shifts, sea-level oscillations, and human activities. In addition to providing new insights into the evolutionary legacy of the genus, the genetic characterizations will be useful for the conservation of *Cycas* species.

## Introduction

Geological events and climate fluctuations during the Pleistocene have profoundly shaped the distribution and speciation of organisms (Hewitt, 2000; Hewitt, 2004). Most tropical species experienced a relatively moderate temperature in the Pleistocene. However, climatic oscillations have also significantly affected the genetic structure, population dynamics, and divergence of many extant global flora and fauna (Pauls et al, 2013; Yang et al., 2016; Mekonnen et al., 2018). Historical processes (e.g., the Ice Age) have left marked imprints on the genetic structure of extant species, especially for long-evolved organisms (Lo´pez-Pujol et al., 2011; Liu et al., 2015). In South China, which is recognized as a biodiversity hotspot, endemic species are currently confronted by dramatic habitat loss due to climate change and anthropogenic activity (Myers et al., 2000; Lo´pez-Pujol et al., 2006; Bax and Francesconi, 2018). Explaining the distribution of endemism plays the important role of understanding historical, evolutionary, and conservation implications of the organisms (Mittermeler, 2017).

Cycads are the oldest and the most primitive group of gymnosperm. They share evolutionary history and morphological characteristics with ferns (spermatophytes with flagella) and gymnosperms (naked seeds) (Zheng et al., 2017). Therefore, cycads are an ideal objective to study the evolutionary history of seed plants. The issues to infer the demographic history of cycads endemic to South China are currently of much interest. Cycads originated before the mid-Permian and reached their greatest diversity during the Jurassic–Cretaceous (Jones, 2002). However, molecular phylogenetic analyses indicated that the extant cycads underwent a synchronous global re-diversification and are not much more than 12 million years old (Nagalingum et al., 2011). In recent years, researchers have studied cycad phylogeny (Liu et al., 2018; Wei et al., 2015), conservation (Feng et al., 2014; Feng et al., 2016; Feng et al., 2017; Gregory and Lopez-Gallego, 2018; Tang et al., 2018a; Zheng et al., 2017), biogeography (Gutiérrez-Ortega et al., 2017; Meerow et al., 2018; Cabrera-Toledo et al., 2019), pollination biology (Tang et al., 2018b; Santiago-Jimnez et al., 2019), and physiology (Vovides and Galicia, 2016). Cycads comprise two families (Cycadaceae and Zamiaceae), with 10 genera and 355 accepted species (Calonje et al., 2019). Unfortunately, 88% of cycads are on the International Union for Conservation of Nature (IUCN) Red List of Threatened Plants (Xiao et al., 2018). *Cycas* L. is the sole genus of the Cycadaceae, and its 117 species are mainly distributed in tropical and subtropical regions of Southeast and East Asia, East Africa, Oceania, and Madagascar (Fragniere et al., 2015). *Cycas* is monophyletic and is sister to all of the other extant cycad genera (Salas-Leiva et al., 2013; Liu et al., 2018). South China is viewed as the origin of *Cycas* (Xiao and Moeller, 2015). *Cycas* species in China are all facing potential endangerment challenges and in need of protection due to the over-collection in wild for the cultivation of commercial plants as well as their ornamental attributes. However, there are still some difficulties in putting the *Cycas* protection action into practice, including the controversial taxonomic status of some *Cycas* species and lack of genetic resources within and among taxa, which is very important for conservation management.

**Figure 1:**
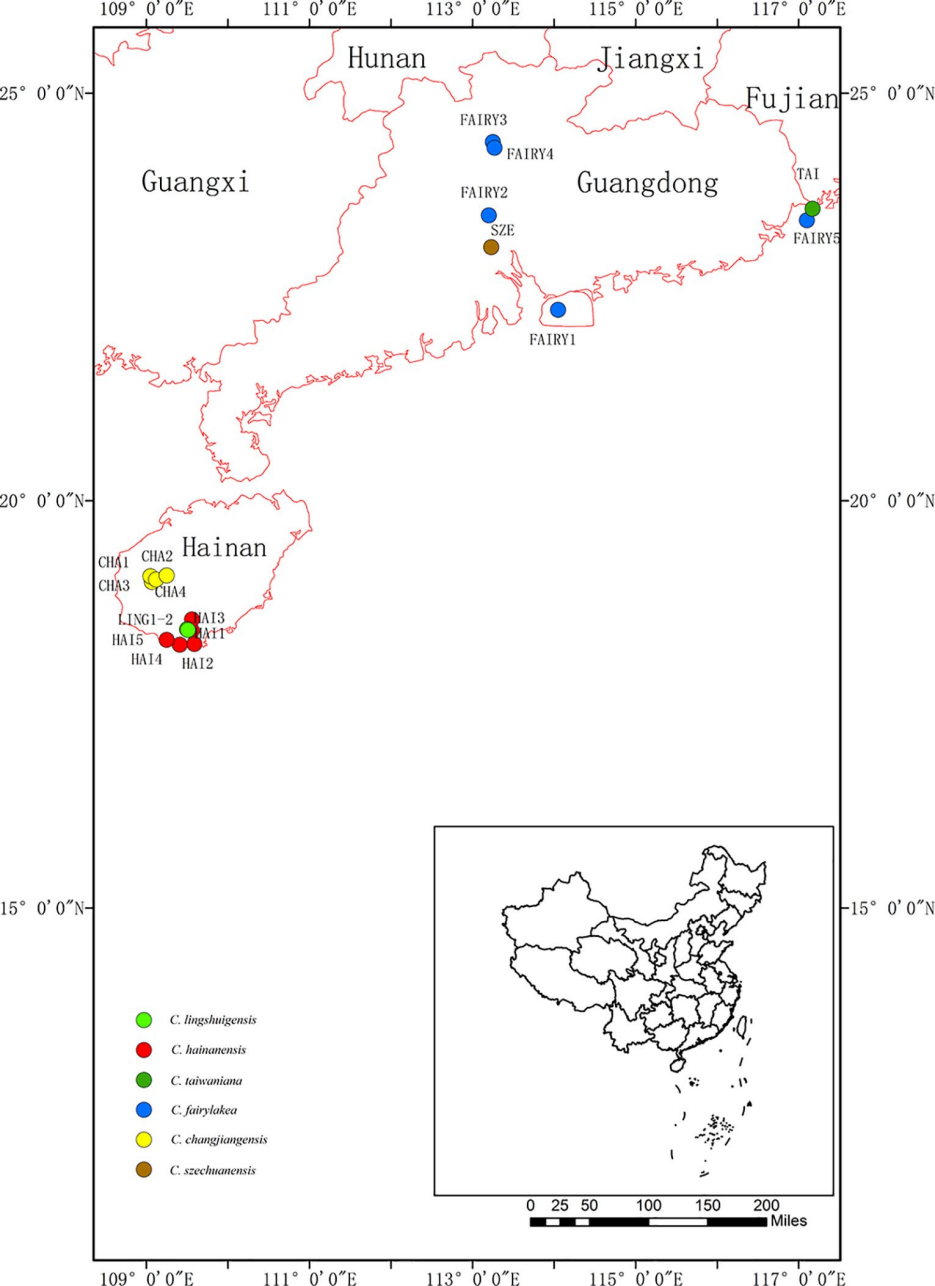
Geographical distribution of 18 populations in the *Cycas taiwaniana* complex. Population codes are explained in **Table 1**.

The *Cycas taiwaniana* complex consists of six morphologically similar and closely related taxa endemic to South China: *Cycas hainanensis* C. J. Chen, *Cycas changjiangensis* N. Liu, *Cycas lingshuigensis* G. A. Fu, *C. taiwaniana* Carruthers, *Cycas szechuanensis* Cheng et L. K. Fu, and *Cycas fairylakea* D. Y. Wang (**Figure 1**). They are mainly distributed in low-/middle-elevation tropical rainforest and hills. Within the complex, *C. hainanensis* C. J. Chen (Cheng et al., 1975), *C. changjiangensis* N. Liu (Liu, 1998), and *C. lingshuigensis* Fu (2004) are endemic to Hainan Island. *C. taiwaniana* Carruthers (Carruther, 1893) has only one wild population, which is in Fujian, and is distinguished by the denticulate margin of its megasporophylls. *C. szechuanensis* Cheng et L. K. Fu (Cheng et al., 1975), which currently occurs in Guangdong and Sichuan provinces, is distinguished from other taxa by its loose megasporophyll cone and its densely dark-brown tomentum in young leaves. *C. fairylakea* D. Y. Wang (Chen and Wang, 1995), which is also distributed in Guangdong, Fujian, differs from *C. szechuanensis* in having a megasporophyll with a distinct apical segment. These species are endangered and their wild populations are dramatically declining due to habitat loss and overexploitation by the ornamental and medical trade. An improved understanding of their population genetic structure and evolutionary history is required for assessing the conservation status of the taxa and for establishing effective management strategies (Funk et al., 2012; Allendorf et al., 2013). Previous studies of the *C. taiwaniana* complex have considered morphological characteristics (Chen and Wang, 1995; Chen and Stevenson, 1999; Wang, 2000; Huang, 2001; Liu and Qin, 2004), geographical distribution (Jian et al., 2005a), karyotype (Nayak et al., 2005), and phylogeography (Nong et al., 2011). Previous studies of their population genetics, however, were based on either limited taxa (Jian et al., 2005b; Jian et al., 2006; Huang et al., 2013a) or insufficient genetic data (Mo et al, 2008). A comprehensive understanding of the population genetics of the *C. taiwaniana* complex based on different molecular markers is needed.

In this study, we used two chloroplast intergenic spacers (chloroplast DNA, cpDNA), two single-copy nuclear genes (SCNGs), and six microsatellite markers (simple sequence repeats, SSR) to explore how the Quaternary climate oscillations may have influenced the current population structure of the *C. taiwaniana* complex. The research had three objectives: 1) to investigate the patterns of genetic variation of the *C. taiwaniana* complex; 2) to examine the historical population demography relative to climate fluctuations; and 3) to reconstruct phylogenetic relationships and estimate divergence time among populations. The genetic data can contribute to the management of the endangered species and can provide insights into the genetic structure and history of the *C. taiwaniana* complex.

## Materials and Methods

### Sampling and DNA Extraction

A total of 354 individuals from six closely related species of the *C. taiwaniana* complex were obtained from 18 populations in Hainan, Guangdong, and Fujian provinces of South China (**Figure 1** and **Table 1**). Fresh leaves were collected in silica gel and stored at −20°C until further processing. Within the 354 samples, 8–11 individuals from each population were randomly selected for chloroplast and single-copy nuclear DNA sequencing. All 354 individuals were used for the microsatellite study. One individual of *Cycas pectinata* was sampled and used as outgroup in the phylogenetic analysis. Total genomic DNA was extracted from dried leaves using a modified cetyltrimethyl-ammonium bromide (CTAB) method (Doyle and Doyle, 1987).

**Table 1:** Locations and sample sizes of 18 populations of the *Cycas taiwaniana* complex surveyed for microsatellites and DNA sequences.

Species	Population code	Location	Longitude	Latitude	Sample size		
					SSR	SCNGs	cpDNA
C. lingshuigensis	LING1	Lingshui, Hainan	109°50′E	18°42′N	20	11	10
G. A. Fu	LING2	Diaoluoshan, Lingshui, Hainan	109°51′E	18°41′N	26	11	9
C. hainanensis	HAI1	Diaoluoshan, Lingshui, Hainan	109°56′E	18°40′N	12	10	10
C. J. Chen	HAI2	Nanwanhou, Lingshui, Hainan	109°59′E	18°24′N	23	10	10
	HAI3	Shenjiecun,Qiongzhong, Hainan	109°56′E	18°54′N	24	10	10
	HAI4	Ganshiling, Sanya, Hainan	109°41′E	18°23′N	23	10	9
	HAI5	Baolongshan, Sanya, Hainan	109°25′E	18°29′N	18	9	10
C. taiwaniana	TAI1	Zhangzhou, Fujian	117°17′E	23°58′N	12	11	10
Carruthers							
C. fairylakea	FAIRY1	Meilin Reservoir, Futian, Shenzhen	114°05′E	22°34′N	23	11	11
D. Y. Wang	FAIRY2	Qingyuan, Guangdong	113°20′E	23°50′N	24	10	10
	FAIRY3	Lechang, Shaoguan, Guangdong	113°25′E	24°40′N	21	10	10
	FAIRY4	Qujiang, Shaoguan, Guangdong	113°27′E	24°33′N	20	11	10
	FAIRY5	Zhaoan, Zhangzhou, Fujian	117°10′E	23°44′N	11	11	10
C.changjiangensis	CHA1	Wangxia, Changjiang, Hainan	109°07′E	19°00′N	15	8	9
N. Liu	CHA2	Dongliu Forest Farm, Bawangling, Hainan	109°05′E	19°07′N	22	9	9
	CHA3	Tuolingfeng, Bawangling, Hainan	109°12′E	19°03′N	22	9	10
	CHA4	Nanbaoshan, Bawangling, Hainan	109°25′E	19°08′N	22	10	10
C. szechuanensis	SZE1	South China Botanical Garden, Guangdong	113°23′E	23°11′N	16	11	10
Cheng et L. K. Fu							
Total	18				354	182	177

### DNA Sequencing and Microsatellite Genotyping

Two chloroplast intergenic spacers (*trn*S-*trn*G and *atp*B-*rbc*L) and two unpublished single-copy nuclear genes (EX929503 and FJ393265) were sequenced for complete analysis (primer information is provided in **Supplementary Table 1**). The two single-copy nuclear genes were developed from the transcriptome between *C. hainanensis* and *C. changjiangensis*. We queried the orthologous pairs with best hits against 959 sets of single-copy nuclear genes shared by *Arabidopsis*, *Populus*, *Vitis*, and *Oryza* (known as APVO genes) (Duarte et al., 2010) using TBLASTN and then designed exon-anchoring and intron-spanning primer using Primer Premier 5. The PCR amplifications for cpDNA were performed in a 35-µl reaction volume containing 2 µl of DNA template(10–40 ng), 3.5 µl of 10× buffer (Takara), 1.75 µl of dNTPs (Takara), 0.35 µl of each primer (forward and reverse), 1.75 µl of MgCl_2_, and 0.35 µl of Taq DNA polymerase (5 U/µl; rTaq, Takara). For SCNGs, the PCR reactions contained 5 µl of DNA template, 0.8 µl of each primer (forward and reverse), 10 µl of 2× Taq Master Mix (Takara), and 3.4 µl of double-distilled water. The PCR amplification of cpDNA began with an initial denaturation of 4 min at 95°C; followed by 35 cycles of 94°C for 45 s, annealing at 53–55°C for 60 s, and 72°C for 90 s; and a final extension at 72°C for 10 min. For SCNGs, the procedure began with an initial denaturation of 4 min at 95°C; followed by 34 cycles of 95°C for 30 s, 55–58°C for 60 s, and 72°C for 60 s; and a final extension at 72°C for 10 min. The product sizes were determined on 1.5% agarose gels in TBE buffer stained with ethidium bromide, and the products were then purified with the Gel Extraction Mini Kit. The PCR products were sequenced using an ABI 3730XL automated sequencer with the BigDye(r) Terminator v3.1 Cycle sequencing kit (Applied Biosystems). The sequences in this study have been deposited in GenBank (accession numbers MK613454-MK613787, and MK620342-MK620685).

Microsatellite markers were selected from developed nuclear microsatellites in *Cycas* (Li et al., 2009; Zhang et al., 2009). Six polymorphic and cross-transferable microsatellites loci were chosen for genotyping (**Supplementary Table 2**). PCR amplifications were carried out in 20 µl reaction volume containing 5 µl of DNA template, 2 µl of 10× buffer (Takara), 0.4 µl of dNTPs (Takara), 0.6 µl of each primer (forward and reverse), 0.2 µl of Taq DNA polymerase (5 U/µl; rTaq, Takara), and 11.2 µl of double-distilled water. The PCR amplification was conducted with the following conditions: an initial denaturation of 4 min at 95°C; followed by 35 cycles of 95°C for 30 s, annealing temperature of 50–56°C for 30 s, and 72°C for 1 min; and a final extension at 72°C for 10 min. PCR products were electrophoretically separated and screened on an ABI 3730XL automated sequencer (Applied Biosystems). The profiles were read using GeneMapper, version 4.0.

### DNA Sequence Analyses

Sequences were edited in SeqMan, version 7.10 (Swindell and Plasterer, 1997) and were multiple aligned with BioEdit, version 7.0.9 (Hall, 1999). Two cpDNA fragments were concatenated with a congruency assessment (Farris et al., 1994) using PAUP* 4.0b10 (Swofford, 2002). For the two SCNGs, heterozygous sites were phased using DnaSP, version 5.0 (Librado and Rozas, 2009). The combined cpDNA matrix and phased SCNGs were used in the following analyses. In addition, cpDNA and SCNG datasets were combined for the phylogenetic analysis.

Nucleotide diversity (*P*_i_) and haplotype diversity (*H*_d_) were calculated with DnaSP, version 5.0 (Librado and Rozas, 2009). Total genetic diversity (*H*_T_), average within-population diversity (*H*_S_), and two parameters of genetic differentiation (*G*_ST_ and *N*_ST_) were estimated using Permut version 2.0 (Pons and Petit, 1996). Permut with 2000 permutations was then used to compare *G*_ST_ and *N*_ST_ at the DNA sequence level. The genetic variation within and between populations was assessed by the hierarchical analysis of molecular variance (AMOVA) with Arlequin, version 3.0 (Excoffier et al., 2005) with 10,000 permutations based on two partitions: 1) populations were grouped according to species and 2) populations were grouped according to distribution regions (Guangdong, Fujian, and Hainan). Haplotype networks were conducted with Network version 4.5 using the median-joining method (Bandelt et al., 1999). Indels were treated as single mutation events.

We inferred the demographic dynamics of the *C. taiwaniana* complex at the gene level by performing three tests. First, we used DnaSP, version 5.0 to examine the following neutrality tests: Fu and Li’s D* and Fu and Li’s F* (Fu, 1997) and Tajima’s *D* (Tajima, 1989) in *F*_S_ value significantly less than zero indicates directional selection. The pairwise mismatch distributions were then examined in DnaSP, version 5.0. Mismatch distribution with a unimodal shape indicates that populations have experienced recent expansion. The sum of squared deviations (SSD) and raggedness index as well as their significance values were calculated using Arlequin, version 3.0. Finally, Bayesian skyline plot (BSP) analyses were performed with BEAST, version 2.3.0 (Drummond and Rambaut, 2007). The Markov chain Monte Carlo (MCMC) analysis was run for 10^8^ generations with sampling for every10^4^ iterations. Tracer, version 1.7 (Rambaut et al., 2014) was used to estimate the effective sample size (ESS). An ESS value greater than 200 indicates acceptable mixing and sufficient sampling. The coalescent Bayesian skyline was selected as priori. The other related parameter settings (evolutionary rates, substitution model, and molecular clock) are specified in the following sections.

### Phylogenetic and Molecular Dating Analyses

Bayesian inference (BI) as implemented in MrBayes, version 3.2.6 (Ronquist et al., 2012) was used to construct a phylogenetic tree of the 18 populations in the *C. taiwaniana* complex with the combined cpDNA–single-copy nuclear DNA (scnDNA) dataset. Prior to combining the sequences, the congruence was examined using the partition–homogeneity test (Farris et al., 1994). The best substitution models for each marker were inferred under the Akaike information criterion (AIC) in MrModeltest, version 3.2 (Nylander, 2004). Furthermore, partition-specific DNA evolution models were used for each gene. Two parallel MCMC analyses started from a random tree and sampled every 1,000 generations for 6 × 10^7^ generations.

Divergence times among populations were estimated using BEAST, version 2.3.0 (Drummond and Rambaut, 2007). A Yule model was set as prior for the tree model. We selected the F81 model with a relaxed uncorrelated molecular clock for combined cpDNAs and an HKY+I model with a strict molecular clock for joint nDNAs. Because an accurate evolutionary rate for *Cycas* was unavailable, we applied the evolutionary rates for seed plants of 1.01 × 10^−9^ substitutions per site per year for synonymous sites for cpDNA and 6.1 × 10^−9^ (5.1–7.1 × 10^−9^) substitutions per site per year for synonymous sites for SCNGs (Graur and Li, 2000; Feng et al., 2017). The MCMC analyses were run for 10^8^ generations and was sampled every 10,000 steps. Convergence of all parameters was checked using Tracer, version 1.7 (Rambaut et al., 2014) with ESS (value >200). The log and tree files from Tracer were combined with LogCombiner. The consensus tree was generated with TreeAnnotator, version 1.8.0 after removal of the first 10% of the trees. The results were visualized with FigTree version 1.4.2 (http://tree.bio.ed.ac.uk/Software/figtree). 

### Microsatellite Data Analyses

Genetic diversity indices were calculated using GenAlex, version 6.5 (Peakall and Smouse, 2012), including the number of alleles (*N*_A_), effective number of alleles (*A*_E_), expected heterozygosity (*H*_E_), observed heterozygosity (*H*_O_), fixation index (*F*), and percentage of polymorphic loci (PPB). Gene flow (Nm) between pairwise populations was estimated based on Nm = (1 − F_ST_)/4 F_ST_ (Wright, 1931), and the differentiation index (F_ST_) was calculated with Arlequin, version 3.0 (Excoffier et al., 2005). Exact tests for deviation from Hardy–Weinberg equilibrium (HWE) were computed in each locus and population using Genepop, version 4.5.1 (Rousset, 2008). AMOVA was conducted in Arlequin, version 3.0 (Excoffier et al., 2005) according to different species and distribution regions. To test for the correlation between genetic distance and geographic distance, the isolated by distance (IBD) model implemented in a mental test (Mantel, 1967) was performed in GenALEx, version 6.3. Genetic distance was calculated with Genepop, version 4.1.4 (Rousset, 2008) according to the formula *F*_ST_/(1 − *F*_ST_). The geographic distance between sampling locations was determined using the R package (Viechtbauer, 2010).

The genetic structure of populations in the *C. taiwaniana* complex was assessed in several ways. First, the individual-based principal coordinate analysis (PCoA) was performed with GenALEx, version 6.5 (Peakall and Smouse, 2012) based on genetic distance between pairs of individuals. Next, unweighted pair group mean analysis (UPGMA) of populations was carried out using NTSYS-pc, version1.4 (Jensen, 1989). Finally, Bayesian model-based clustering analysis of the 18 populations was conducted using STRUCTURE, version 2.2 (Pritchard et al., 2000). The number clusters (*K*) from *K* = 1 to 18 were run 20 times. Each run contained a burn-in of 10^5^ iterations and 10^5^ replications of MCMC. The most likely number of *K* was estimated in Structure Harvest, version online, version 0.6.9 (Earl and Holdt, 2012) using Δ*K* (Evanno et al., 2005), where the modal value of the distribution is located at the real *K*. The STRUCTURE results were plotted with DISTRUCT (Rosenberg, 2004).

We performed bottleneck effect test of 18 populations in the *C. taiwaniana* complex to investigate demographic dynamic. The two-phased model (TPM) implemented with Wilcoxon test method was selected in bottleneck analysis. We also tested for a bottleneck by using a mode shift model (Piry et al., 1999) in BOTTLENECK, version 1.2.02 (Cornuet and Luikart, 1996).

## Results

### Genetic Diversity and Differentiation

The combined and aligned cpDNA was 1,594 bp long (877 bp for *trn*S-*trn*G and 717 bp for *atp*B-*rbc*L) with a rate of homogeneity (*P* = 0.08, > 0.05). A total of 17 polymorphic sites and nine haplotypes (H1–H7) across 177 individuals were identified (**Supplementary Table 3**). For cpDNA, haplotype diversity was detected only in populations FAIRY5, CHA1, CHA2, CHA3, and CHA4. Population CHA1 of *C. changjiangensis* had the highest haplotype diversity (*H*_d_ = 0.64), and CHA2 had the highest nucleotide diversity (*P*_i_ = 0.0011) (**Supplementary Table 3**). The average within-population diversity (*H*_S_ = 0.095) was much lower than the total diversity (*H*_T_ = 0.772) (**Table 2**). *N*_ST_ was significantly larger than *G*_ST_, suggesting that haplotype similarities were correlated with geographic distribution (**Table 2**). The hierarchical AMOVA (**Supplementary Table 3**) indicated that the genetic variations were mainly due to species differences (87.97%) and interregional differences (80.02%), suggesting a regional population substructure.

**Table 2:** Genetic diversity and genetic differentiation of the combined chloroplast DNA (cpDNA) and single-copy nuclear DNA in all populations of the *Cycas taiwaniana* complex.

Marker	*H*_S_	*H*_T_	*G*_ST_	*N*_ST_	*U* test
cpDNA	0.095	0.772	0.877	0.952	*N*st > *G*st**
EX	0.798	0.982	0.187	0.466	*N*st > *G*st**
FJ	0.622	0.902	0.248	0.536	*N*st > *G*st**

**P < 0.01.

The aligned SCNGs EX and FJ were 941 and 784 bp long, respectively. EX and FJ had eight and three recombination events, respectively. Two SCNGs showed variable genetic diversity. In EX, 56 haplotypes were obtained based on 33 polymorphism sites (**Supplementary Table 3**). At the species level, *C. lingshuigensis* had the highest genetic diversity (*H*_d_ = 0.93, *P*_i_ = 0.0045). For FJ, 29 haplotypes with 32 polymorphism loci were identified (**Supplementary Table 3**). *C. changjiangensis* had the highest species-scale haplotype and nucleotide diversity (*H*_d_ = 0.86, *P*_i_ = 0.0034). The overall estimated genetic diversity was larger for EX (*H*_d_ = 0.94, *P*_i_ = 0.0048) than for FJ (*H*_d_ = 0.83, *P*_i_ = 0.0028). The total genetic diversity in both EX and FJ was high. *N*_ST_ was significantly larger than *G* *_ST_*, indicating a phylogeographic structure of haplotype distribution (**Table 2**). The difference among species explained 28.69% of the EX variation and 32.48% of the FJ variation. Among species, variations were higher within populations (50.44% for EX and 49.43% for FJ) than among populations (20.87% for EX and 18.09% for FJ). Among regions, higher variances (49.16% for EX and 46.76% for FJ) were also distributed within populations than among populations based on SCNG data. The values of *F*_ST_ ranged from 0.330 to 0.962, indicating a highly significant genetic differentiation among populations in the *C. taiwaniana* complex (**Table 3**).

**Table 3:** Analysis of molecular variance (AMOVA) of the *Cycas taiwaniana* complex based on chloroplast DNA (cpDNA), single-copy nuclear genes (SCNGs), and microsatellites.

Marker	Source of variation	*d.f.*	Sum of squares	Variance components	Percentage of variation	*F*_ST_
cpDNA	Among species	5	303.543	2.71079	87.97	
	Among populations	11	28.766	0.25461	8.26	0.916***
	Within populations Among regions Among populations Within populations	150 2 14 150	17.433 303.543 91.575 17.433	0.11622 3.09236 0.65579 0.11622	3.77 80.02 16.97 3.01	0.962***
EX	Among species	5	247.690	0.69780	28.69	
	Among populations	11	124.730	0.50767	20.87	0.496***
	Within populations Among regions Among populations Within populations	327 2 14 327	401.237 126.259 246.161 401.237	1.22702 0.45472 0.81434 1.222702	50.44 18.22 32.62 49.16	0.508***
FJ	Among species	5	127.415	0.37926	32.48	
	Among population	11	52.635	0.21125	18.09	0.506***
	Within populations	327	188.767	0.57727	49.43	
	Among regions	2	79.621	0.32876	26.63	
	Among populations	14	100.429	0.32842	26.60	0.532***
	Within populations	327	188.767	0.57727	46.76	
SSR	Among species	5	267.733	0.36101	16.96	
	Among populations Within populations Among regions Among populations Within populations	12 690 2 150 690	181.498 983.630 166.598 282.633 983.630	0.34247 1.42555 0.38169 0.43554 1.42555	16.09 66.96 17.02 19.42 63.56	0.330*** 0.364***

d.f. degrees of freedom.

***P < 0.001.

A total of 117 alleles ranging from 16 to 24 per locus were identified based on SSR across the 354 individuals (**Supplementary Table 4**). At the species level, *C. changjiangensis*, *C. hainanensis*, and *C. lingshuigensis* had high genetic diversity according to the values of the genetic parameters, *N*_A_ and *A*_E_ (**Supplementary Table 5**). The average observed heterozygosity (*H*_O_) and expected heterozygosity (*H*_E_) across all populations were 0.620 and 0.565, respectively (**Table 4**). The values for the fixation index (*F*) were negative in populations HAI2, TAI1, FA*I*RY1, FAIRY2, FAIRY3, FAIRY4, FAIRY5, CHA3, and SZE1, suggesting that these nine populations were deviated from HWE (**Table 4**). Although the population pairs of *Cycas hainaniana* with *C. lingshuigensis* and *C. hainaniana* with *C. changjiangensis* had a higher level of gene flow (**Supplementary Table 6**), the gene flow (Nm) between pairs of populations was usually <1. Of the 108 population–locus comparisons, 42 significantly deviated from HWE (**Supplementary Table 7**). The AMOVA revealed that 66.96% and 63.56% of variation were attributed to differences among individuals within populations at the species and regions levels, respectively (**Table 3**). Genetic distance was significantly correlated with geographic distance (*r* = 0.421, *P* = 0.001), supporting an overall pattern of IBD in the *C. taiwaniana* complex (**Supplementary Figure 2**).

**Table 4:** Genetic diversity parameters within populations of the *Cycas taiwaniana* complex based on microsatellites.

Population	*N*_A_	*A*_E_	*H*_O_	*H*_E_	*F*	PPL (%)
LING1	9.833	5.953	0.792	0.826	0.043*	100.00
LING2	9.500	5.191	0.721	0.767	0.060**	100.00
HAI1	7.333	4.477	0.639	0.760	0.162*	100.00
HAI2	6.333	3.800	0.804	0.725	−0.116***	100.00
HAI3	7.167	4.283	0.660	0.740	0.114*	100.00
HAI4	7.333	4.312	0.629	0.707	0.114***	100.00
HAI5	7.667	3.863	0.519	0.679	0.236***	100.00
TAI1	1.833	1.833	0.833	0.417	−1.000***	83.33
FAIRY1	2.333	1.475	0.268	0.263	−0.021*	83.33
FAIRY2	1.333	1.333	0.333	0.167	−1.000***	33.33
FAIRY3	1.667	1.649	0.627	0.329	−0.904***	66.67
FAIRY4	1.667	1.517	0.517	0.266	−0.763***	66.67
FAIRY5	1.667	1.656	0.636	0.331	−0.923***	66.67
CHA1	5.500	3.448	0.583	0.643	0.160***	100.00
CHA2	8.167	4.620	0.687	0.719	0.042***	100.00
CHA3	6.333	3.938	0.773	0.737	−0.055***	100.00
CHA4	7.167	3.990	0.568	0.717	0.232***	100.00
SZE1	2.667	1.762	0.563	0.376	−0.220***	100.00
Mean	5.306	3.283	0.620	0.565	−0.134	88.89

N_a_, number of alleles; N_e_, number of effective alleles; H_O_, observed heterozygosity; H_E_, expected heterozygosity; F fixation index; PPL percentage of polymorphic loci.

*P < 0.05, **P < 0.01, ***P < 0.001.

### Haplotype Network and Genetic Clustering

**Figure 2:**
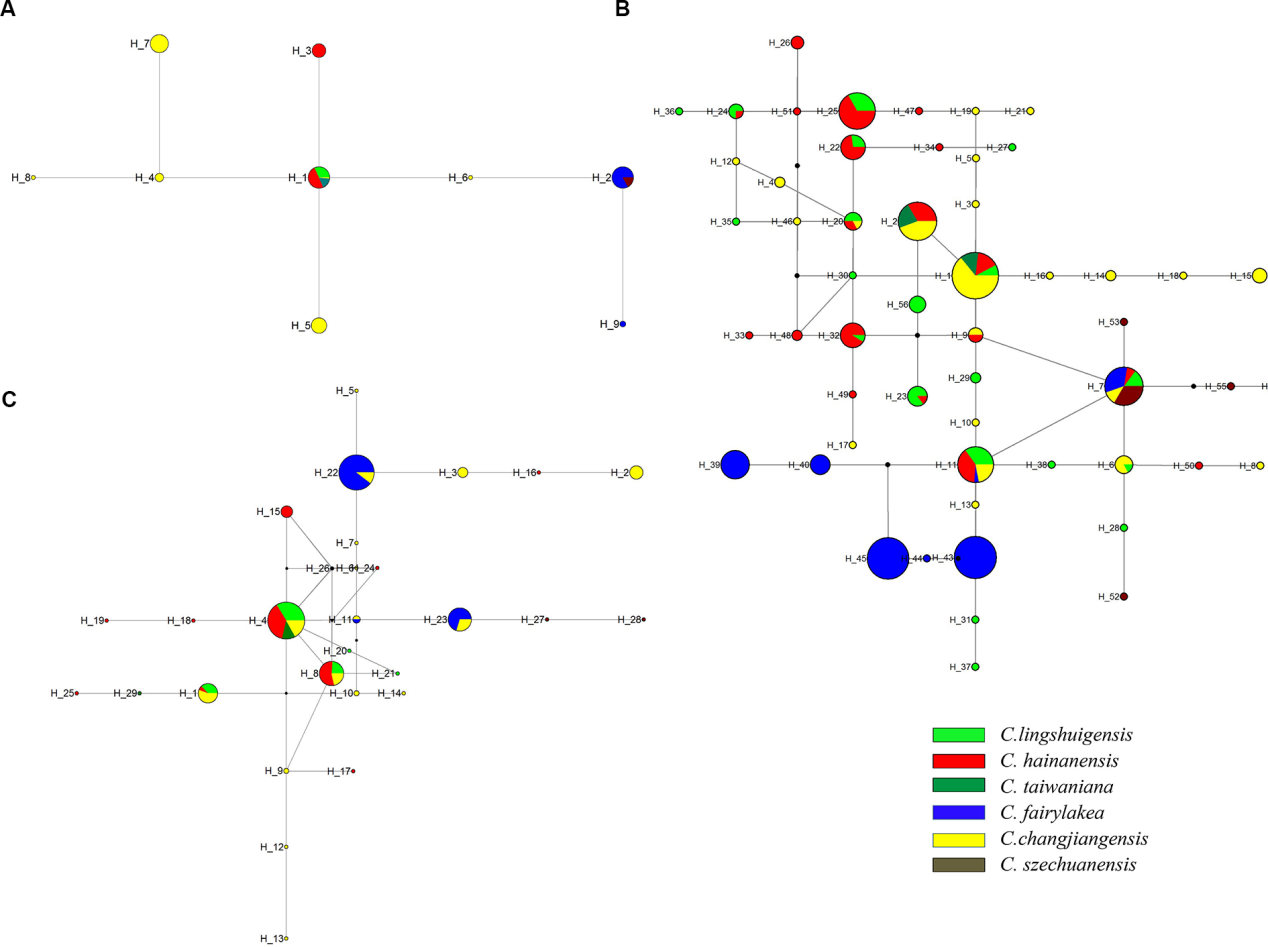
Haplotype network of the *Cycas taiwaniana* complex based on chloroplast DNA (cpDNA) **(A)**, EX **(B)**, and FJ **(C)**. The *small black dots* represent missing intermediate haplotypes. Each indicates one haplotype. The diameter of the *circle* is proportional to the number of samples. Each of the six species of the *C. taiwaniana* complex is represented by a *different color*.

The cpDNA network indicated that H1 was the most common and widespread haplotype in seven populations across four species and is candidate ancestral haplotype. H2 was the second most frequent haplotype, which was found in six populations across two species. The remaining haplotypes occurred in only one species (**Figure 2A**). For EX, haplotype H1 was the most frequently shared in four species and was located in the interior position of the network, suggesting that it might be an ancestral haplotype (**Figure 2B**). For FJ, H4 was the most widely shared haplotype. However, haplotype relationship remained ambiguous due to the presence of multiple “loops” (**Figure 2C**).

**Figure 3:**
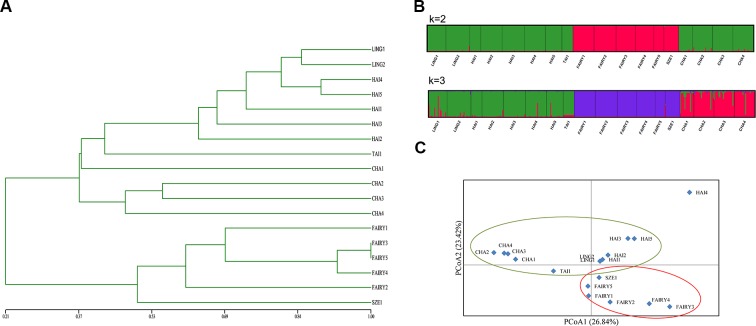
Clusters within the *Cycas taiwaniana* complex. **(A)** An unweighted pair-group method with arithmetic averages (UPGMA) phenogram. **(B)** Bayesian inferences (*K* = 2 and *K* = 3). (C) Principal coordinates analysis (PCoA) of SSR phenotypes from 18 populations of 354 individuals of the *C. taiwaniana* complex. The percentage of variation attributed to each axis is indicated.

The UPGMA dendrogram for microsatellite data demonstrated that populations FAIRY1, FAIRY2, FAIRY3, FAIRY4, and SZE1 grouped into one clade and that the other populations formed a second clade (**Figure 3A**). Structure analysis (**Figure 3B**) supported two genetic clusters based on Δ*K* statistics (**Supplementary Figure 1**). The optimal *K* value was 2, indicating that the 18 populations from the *C. taiwaniana* complex were separated into two clusters. The second fittest value (*K* = 3) indicated that the populations were grouped into three clades. However, there were some admixtures when the *K* value was 3. Low genetic composition was detected among *C. lingshuigensis*, *C. hainanensis*, *C. taiwaniana*, and *C. changjiangensis*. The UPGMA and structure analyses were supported by the PCoA (**Figure 3C**).

### Historical Demography

Regarding cpDNA data, we only conducted mismatch analyses for the three species that had haplotype genetic diversity: *C. changjiangensis* (**Supplementary Figure 3A**), *C. fairylakea* (**Supplementary Figure 3B**), and *C. hainanensis* (**Supplementary Figure 3C**). The mismatch analysis of cpDNA revealed a unimodal distribution in *C. hainanensis* (**Supplementary Figure 3C**), suggesting a recent population expansion. This inference was supported by the significant negative values of Fu and Li’s D* and F* (**Supplementary Table 8**). For SCNGs, *C. taiwaniana* showed significant positive values for Fu and Li’s F* and for Tajima’s *D*, suggesting a stable demographic history. Furthermore, multimodal distribution patterns were detected in *C. taiwaniana*, which indicated that the species has not undergone recent population expansion (**Supplementary Figures 3I, K**). For EX, multimodal distribution patterns were also detected in C. *fairylakea*, C. *hainanensis*, C. *lingshuigensis*, C. *szechuanensis and* C. *changjiangensis* (**Supplementary Figure 3D–H**). For FJ, C. *szechuanensis*, C. *fairylakea* and C. *changjiangensis* showed multimodal distribution patterns (**Supplementary Figure 3J, L and M**). However, unimodal distributions were detected in C. *hainanensis* and C. *lingshuigensis* (**Supplementary Figure 3N and O**).

**Figure 4:**
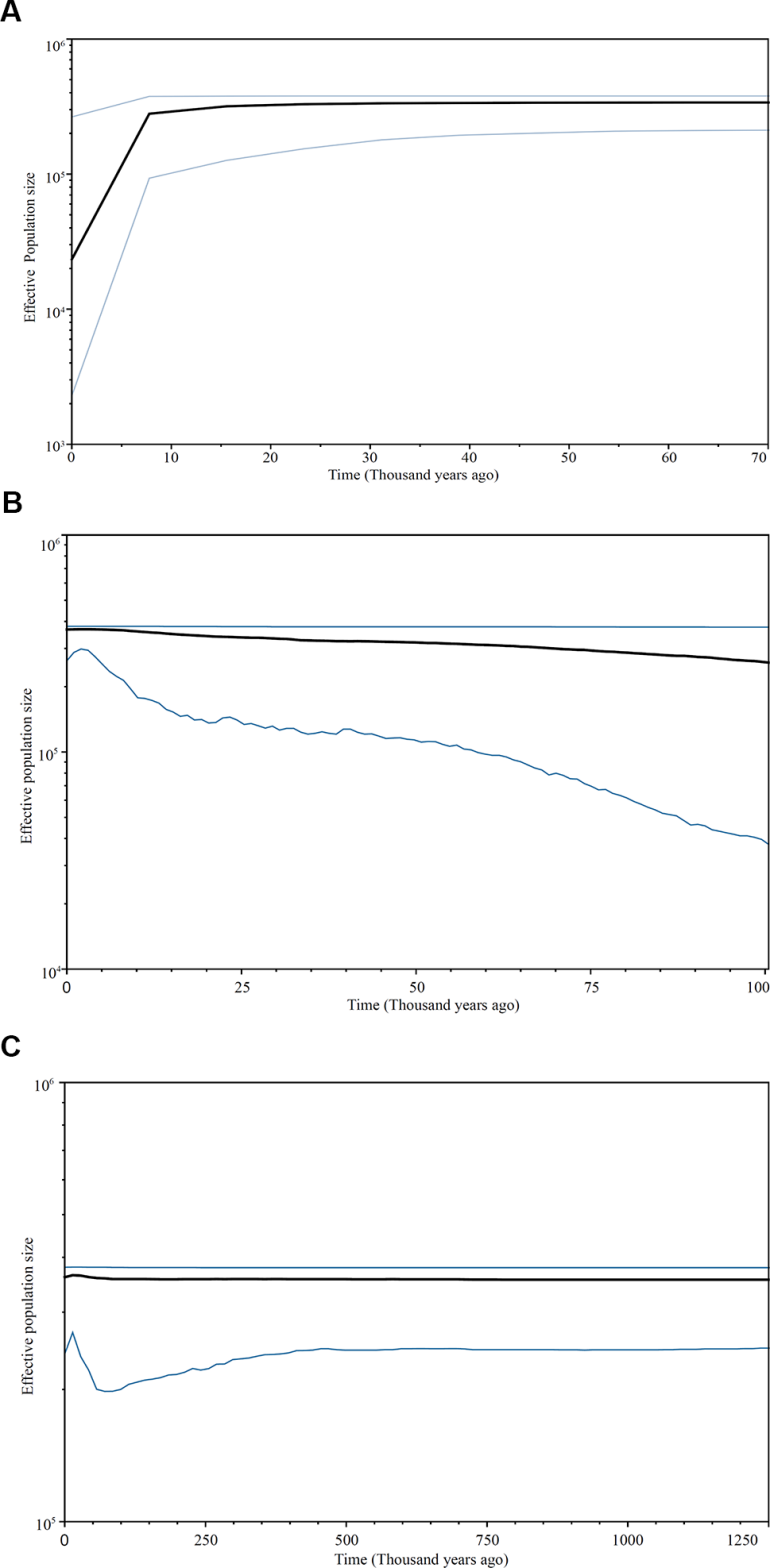
Bayesian skyline plots based on cpDNA **(A)** and single-copy nDNA: EX **(B)** and FJ **(C)**. The *X*-axis indicates time in thousands of years ago and the *Y*-axis represents the effective size multiplied by generation time on a log scale. The *black line* indicates the estimated median and the area *between the blue lines* represents the 95% confidence interval.

The Bayesian skyline plot analyses revealed a complex demographic history in the *C. taiwaniana* complex. The results derived from cpDNA, EX, and FJ were inconsistent. Based on cpDNA data, the population size experienced a long history of equilibrium and rapid contraction since approximately 8,000 years ago (**Figure 4A**). For EX, slow population growth with a subsequent slight decline since 5,000 years ago was detected (**Figure 4B**). The Bayesian skyline plot of FJ indicated three dynamic events: the *C. taiwaniana* complex was stabile from 1.25 to 0.07 Ma, subsequently experienced a population expansion beginning about 0.07 Ma, and then underwent a slow decline from 0.015 Ma to the present (**Figure 4C**).

According to the bottleneck analysis, four populations (HAI3, TAI1, FAIRY3, and FAIRY5) had a significant excess of heterozygosity and deviated from mutation-drift equilibrium (**Supplementary Table 9**). In addition, the mode shift tests revealed that most of the populations had a normal L-shaped distribution, except for populations TAI1, FAIRY2, FAIRY3, FAIRY4, and FAIRY, which suggested that only these four populations had experienced a severe bottleneck (**Supplementary Table 9**).

### Phylogenetic Relationship and Divergence Time

**Figure 5:**
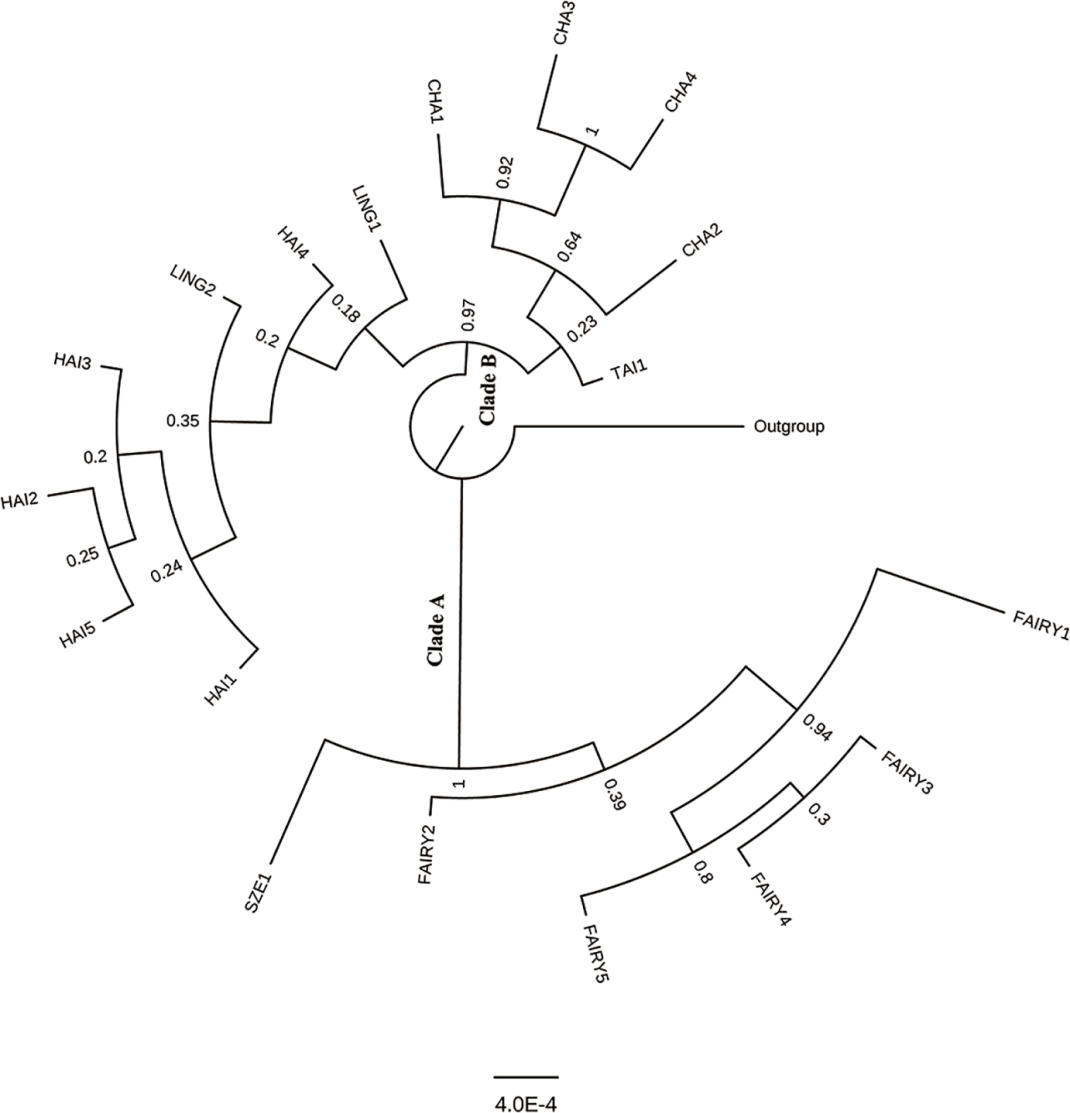
Phylogenetic relationships among 18 populations in the *Cycas taiwaniana* complex inferred from combined two single-copy nuclear genes and two chloroplast intergenic spacers based on Bayesian inference.

The partition–homogeneity test analysis indicated that the cpDNA and scnDNA sequences could be combined for phylogenetic analysis (*P* = 0.1). The phylogenetic tree constructed from the concatenated cpDNA–scnDNA dataset was partly unresolved with *C. pectinata* as the outgroup (**Figure 5**). However, two main clades in the *C. taiwaniana* complex were obtained with high support: one clade comprised *C. fairylakea* and *C. szechuanensis* (clade A), and the second clade (clade B) comprised *C. hainaniana*, *C. lingshuigensis*, *C. changjiangensis*, and *C. taiwaniana* (**Figure 5**). Within the first clade, *C. szechuanensis* was placed as the sister group of *C. fairylakea* with robust support. The relationships among *C. hainaniana*, *C. lingshuigensis*, *C. changjiangensis*, and *C. taiwaniana* were weakly supported.

The species trees for cpDNA and SCNG datasets had similar topologies and corroborated the presence of two well-supported clades (**Supplementary Figure 4**). The populations based on cpDNA sequences were divided into two lineages that diverged at approximately 1.790 Ma (95% HPD = 0.85–2.87 Ma) (**Supplementary Figure 4A**). For SCNGs, populations also clustered into two lineages that diverged about 0.311 Ma (95% HPD = 0.11–0.71 Ma) (**Supplementary Figure 4B**). Dating analyses suggested that six taxa of the *C. taiwaniana* complex diverged in the Middle Pleistocene.

## Discussion

### Genetic Diversity and Differentiation

Population size, life history traits, breeding, and eco-landscape can affect genetic variation within or among species (Nybom, 2004; Reisch and Bernhardt-Roemermann, 2014; Reisch et al., 2017). Most Asian *Cycas* are narrowly distributed and have similar life history traits, such as dioecy, long life spans, anemophilous, or entomophilous (Feng et al., 2014). At the species level, *C. fairylakea* had lower genetic diversity based on cpDNA and SSR than other Asian *Cycas* species (Zheng et al., 2017). *C. changjiangensis* had high genetic diversity based on cpDNA and SCNGs, which is consistent with previous results based on allozyme analysis by Jian et al. (2005c). The total genetic diversity of the *C. taiwaniana* complex among all populations was higher than the mean value (*H*_T_ = 0.67) of 170 plant species (Petit et al., 2005). Probable explanations for the high genetic variation include retention of ancestral polymorphisms (Yang et al., 2015), refugia (Feng et al., 2014; Lin et al., 2018), genetic drift (Van et al., 2010), or genome accumulation during a long evolutionary history (Feng et al., 2014).

In the current study of 18 populations in the *C. taiwaniana* complex, the total genetic diversity (*H*_T_ = 0.772 based on cpDNA) was much larger than the average within-population diversity (*H*_S_ = 0.095), suggesting that the level of genetic differentiation among populations is high (*G*_ST_ = 0.977 and *N*_ST_ = 0.952). In addition, N_ST_ was significantly greater than *G*_ST_, which indicates the presence of a distinct phylogeographical structure in the *C. taiwaniana* complex. The *F*_ST_ values obtained by cpDNA were also much greater than the average value of 0.67 previously estimated from other maternally inherited markers (Petit et al., 2005), indicating significant genetic differentiation among populations. SCNG- and SSR-based *F*_ST_ values greater than 0.25 also demonstrate significant genetic differentiation (Wright, 1978). In *Cycas* species, the cpDNA is maternally inherited by seed, whereas SCNG is biparentally inherited through seed flow and pollen flow. In this study, the higher *F*_ST_ detected by cpDNA than those in SCNG and SSR suggested that the pollen flow of *C. taiwaniana* complex was greater than the seed flow (Gong and Gong, 2016; Xiao et al., 2018).

### Phylogeographical Pattern

In the STRUCTURE analysis, the low genetic admixture and robust population clusters also suggest high genetic differentiation. The *Cycas* species, with similar life history and reproductive characteristics, are thought to have a high level of genetic variation within populations and significant genetic differentiation among populations (Liu et al., 2015). The significant genetic differentiation and clear genetic structure observed in the *C. taiwaniana* complex may be explained by limited gene flow (Feng et al., 2014; James et al., 2018), which had also been reported for other *Cycas* species (Zhao and Gong, 2015; Feng et al., 2017). The effective propagation distance of *Cycas* species is 2–7 km (Yang and Meerow, 1996). The seeds of *Cycas* species are too large to disperse for a long distance and drop near the mother plants, enhancing the probability of inbreeding (Hall and Walter, 2011).

Our STRUCTURE analysis of SSR detected two distinct clusters, which correspond to the morphological characteristics and geography. The two lineages were also supported by a phylogenetic tree, UPGMA and PCoA clustering. *C. szechuanensis* and *C. fairylakea* from inland China formed one clade, whereas *C. hainanensis*, *C. changjiangensis*, and *C. lingshuigensis* from Hainan Island formed the other clade. There was a low level of admixture for *Cycas* species between Hainan Island and the Mainland. Island populations that were isolated from Mainland relatives could exhibit genetic divergence, local adaptation, and incipient speciation. Furthermore, the limited dispersal abilities of *Cycas* on islands and their adaptation to the local environments are likely to produce distinct lineages (Huang et al., 2013b). However, *C. taiwaniana* with only one population from Fujian were clustered into Hainan Island clade. The exact origin of *C. taiwaniana* remains unclear. If the *C. taiwaniana* was ever part of the flora of Mainland China, it is probably now extinct in that habitat. The *C. taiwaniana* complex is dioecious and allogamous. Their seeds are heavy and contain the toxic compound, which hampers long distance disposal by water or animals (Schneider et al., 2002). Based on our BEAST analysis, *C. taiwaniana* diverged more recently than *C. hainanensis*, *C. changjiangensis*, and *C. lingshuigensis* from Hainan Island (**Supplementary Figure 4**). Therefore, *C. taiwaniana* is likely originated on Hainan Island, then migrated to the Mainland and survived in Fujian now.

Both the BI analysis from the combined cpDNA–SCNGs dataset and the two Bayesian species trees revealed similar relationships among the two major lineages within the *C. taiwaniana* complex. The results presented here are mostly consistent with the recent molecular phylogeny of *Cycas* (Liu et al., 2018). Clade A (*C. szechuanensis* and *C. fairylakea*) is well supported with PP = 1. The other clade B (*C. hainanensis*, *C. changjiangensis*, *C. lingshuigensis*, and *C. taiwaniana*) located in Hainan Island and Fujian Province is supported by PP = 0.97. However, the relationship within *C. hainanensis*, *C. changjiangensis*, *C. lingshuigensis*, and *C. taiwaniana* is not very clear. The clade including species from Hainan Island is geographically isolated from species from South China, indicating that limited genetic exchange may have promoted the divergence of the two clades. Hainan Island was separated from Mainland China by the Qiongzhou Strait, which formed approximately 2.5–25 million years ago (Wu et al., 2012). The land bridge of Qiongzhou Strait has experienced cyclic upheaval and submergence with sea-level changes (Zhao et al., 2007). The low level of gene exchange between island taxa and continental relatives might have been facilitated by the periodic presence and absence of the land bridge (Zhao et al., 2013).

### Divergence Time and Demographic Dynamics

Phylogenetic analyses revealed two lineages (A and B) in *C. taiwaniana* complex. The divergence time of the two lineages based on cpDNA and SCNG sequences is about 1.790 and 0.311 Ma, respectively, corresponding to a period of glacial cycle during the Middle Pleistocene, which verifies recent divergence in the former research (Nagalingum et al., 2011). The divergence time from cpDNA and SCNG was inconsistent, which is caused by different evolutionary rates and migration modes between organelle (pollen migration) and nuclear markers (pollen migration and seed migration; seed migration is very little) (Wolfe et al., 1987; Zheng et al., 2016). The recent divergence between the two lineages indicated that the species in clade B colonized Hainan Island in the Middle Pleistocene when land bridges formed (Shi et al., 2006; Huang et al., 2013b). A compilation of molecular data for divergence in subspecies and species complexes showed that species were formed through the Pliocene and Pleistocene (Hewitt, 1993). We suspect that the geographic isolation and low seed dispersal ability in the *C. taiwaniana* complex have generated genetic isolation among populations, i.e., have promoted a “terrestrial island speciation” (Wang et al., 2017).

Although the Pleistocene ice events were evident in Europe (Taberlet et al., 1998), the climate also changed in China (Harrison et al., 2001). Climate oscillations during the Pleistocene had effects on the demographic history of plants (Dorsey et al., 2018; Zhang et al., 2019). Different *Cycas* species had different population dynamics to respond to glacial and interglacial influences. *Cycas debaoensis* (Zhan et al., 2011), *Cycas simplicipinna* (Feng et al., 2014), *Cycas multipinnata* (Gong et al., 2015), and *Cycas diannanensis* (Liu et al., 2015) have experienced population contractions, while *Cycas revoluta* and *Cycas taitungensis* (Chiang et al., 2009) have expanded their geographical distributions. In this study, a recent population contraction in the *C. taiwaniana* complex is suggested based on three markers (cpDNA, SCNGs, and SSR). It is possible that refugia, temperature changes, and recent human activities caused the contractions in the *C. taiwaniana* complex.

### Implications for Conservation

To successfully protect threatened species, it is necessary to preserve the maximum genetic diversity (Montalvo et al., 1997; Jump and Penuelas, 2005). Species with unique haplotypes should be given the highest priority protection (Petit et al., 1998). In this study, we found a higher genetic diversity in the *C. taiwaniana* complex than many other *Cycas* species in China. However, severe habitat loss and illegal exploitation for trading and ornament have severely threatened the existing populations of *C. taiwaniana* complex. *C. hainanensis*, *C. changjiangensis*, *C. lingshuigensis*, and *C. taiwaniana* are regarded as endangered (EN), while *C. szechuanensis* and *C. fairylakea* are critically endangered (CR) species (IUCN, Species Survival Commission, Cycad Specialist Group, Source: https://www.iucnredlist.org/search). Therefore, we proposed to protect all the existing wild populations of *C. taiwaniana* complex and their habitats *in situ*. Some species and populations with unique haplotypes, such as *C. hainanensis*, *C. changjiangensis*, *C. lingshuigensis*, *C. szechuanensis*, *C. fairylakea*, and populations LING1, HAI2, FAIRY1, FAIRY5, and CHA1-4 should be given the highest priority protection. We also recommend the *ex situ* conservation for those critically endangered species (*C. szechuanensis* and *C. fairylakea*) and important populations (such as small population size or with unique haplotypes) by collecting seeds or seedlings from wild (Griffith et al., 2015; Griffith et al., 2017). Furthermore, conservation awareness of local people and law establishment by the government should be improved to protect *C. taiwaniana* complex from extinction.

## Conclusion

Our findings demonstrate that the *C. taiwaniana* complex has a high level of genetic diversity and a high degree of genetic differentiation among populations. Our findings also suggest that the *C. taiwaniana* complex have two main lineages. *C. fairylakea* was incorporated into *C. szechuanensis* and the other populations merged into one lineage. The genetic structure of the *C. taiwaniana* complex has been greatly affected by Pleistocene climate shifts, sea-level oscillations, and human activities, which may increase our ability to identify the phylogeographic patterns in these species and to detect other potential evolutionary lineages within the *C. taiwaniana* complex across South China. In addition to providing insight into the evolution of *Cycas* species, the findings of this study will be useful for the conservation of these endangered plants.

## Data Availability Statement

The datasets generated for this study can be found in the GenBank with accession numbers MK613454-MK613787, MK620342-MK620685.

## Author Contributions

S-GJ designed the research and collected the study materials. X-HW performed the SCNG sequencing, data analyses, and wrote the manuscript. JL and L-MZ contributed to material collection, DNA extraction, SSR and cpDNA sequencing. S-GJ, Q-MM, Z-WH, and XG contributed to manuscript revisions. All authors approved the final manuscript.

## Funding

This work was supported by the National Key Research and Development Program of China (2016YFC0503104) and the National Natural Science Foundation of China (grant no. 31070304).

## Conflict of Interest

The authors declare that the research was conducted in the absence of any commercial or financial relationships that could be construed as a potential conflict of interest.

## References

[B1] Allendorf F. W. Luikart G. Aitken S. N. (2013). Conservation and the genetics of populations (Chichester: John Wiley and Sons).

[B2] Bandelt H. J. Forster P. Rohl A. (1999). Median-joining networks for inferring intraspecific phylogenies. Mol. Biol. Evol. 16, 37–48. 10.1093/oxfordjournals.molbev.a026036 10331250

[B3] Bax V. Francesconi W. (2018). Conservation gaps and priorities in the Tropical Andes biodiversity hotspot: Implications for the expansion of protected areas. J. Environ. Manage. 232, 387–396. 10.1016/j.jenvman.2018.11.086 30500702

[B4] Cabrera-Toledo D. Gonzalez-Astorga J. Vovides A. P. Casas A. Vargas-Ponce O. Carrillo-Reyes P. (2019). Surviving background extinction: Inferences from historic and current dynamics in the contrasting population structures of two endemic Mexican cycads. Popul. Ecol. 61, 62–73. 10.1002/1438-390x.1008

[B5] Calonje M. Stevenson D. W. Osborne R. (2013-2019). The World List of Cycads, online edition [Internet]. [cited 2019 Mar 07]. Available from: http://www.cycadlist.org.

[B6] Carruther W. (1893). *Cycas taiwaniana* sp. nov. and *C. seemanii*A. Br. J. Bot. 31, 1–3.

[B7] Chen J. R. Stevenson D. W.Cycadaceae (1999). Flora of China. Eds. Wu Z. Y. Raven P. H. (Beijing and Saint Louis: Science Press and Missouri Botanieal Garden Press), 1–7. Pp. 1-7 in:.

[B8] Chen J. R. Wang Y. Z. (1995). Taxonomy of Cycas L.(Cycadaceae) in China. Bull. Bot. 12, 14–20.

[B9] Cheng W. C. Fu L. K. Cheng C. Y. (1975). Gymnospermae sinicae. Acta Phytotax. 13, 81–82.2. 10.3724/SP.J.1002.1975

[B10] Chiang Y. C. Hung K. H. Moore S. J. Ge X. J. Huang S. Hsu T. W. (2009). Paraphyly of organelle DNAs in *Cycas* Sect. Asiorientales due to ancient ancestral polymorphisms. BMC Evol. Biol. 9, 161–180. 10.1186/1471-2148-9-161 19589178 PMC3224665

[B11] Cornuet J. M. Luikart G. (1996). Description and power analysis of two tests for detecting recent population bottlenecks from allele frequency data. Genet. 144 (4), 2001–2014. 10.0000/PMID8978083 PMC1207747 8978083

[B12] Dorsey B. L. Gregory T. J. Sass C. Specht C. D. (2018). Pleistocene diversification in an ancient lineage: a role for glacial cycles in the evolutionary history of Dioon Lindl. (Zamiaceae). Am. J. Bot. 105, 1512–1530. 10.1002/ajb2.1149 30229556

[B13] Doyle J. J. Doyle J. L. (1987). A rapid DNA isolation procedure for small quantities of fresh leaf tissue. Phytochem. Bull. 19, 11–15.

[B14] Drummond A. J. Rambaut A. (2007). BEAST: Bayesian evolutionary analysis by sampling trees. BMC Evol. Biol. 7, 214–222. 10.1186/1471-2148-7-214 17996036 PMC2247476

[B15] Duarte J. M. Wall P. K. Edgeer P. P. Landherr L. L. Ma H. Pires J. C. (2010). Identification of shared single copy nuclear genes in *Arabidopsis*, *Populus*, *Vitis* and *Oryza* and their phylogenetic utility across various taxonomic levels. BMC Evol. Biol. 10, 61–78. 10.1186/1471-2148-10-61 20181251 PMC2848037

[B16] Earl D. A. Holdt B. M. (2012). STRUCTURE HARVESTER: a website and program for visualizing STRUCTURE output and implementing the Evanno method. Conserv. Genet. Resour. 4, 359–361. 10.1007/s12686-011-9548-7

[B17] Evanno G. Regnaut S. Goudet J. (2005). Detecting the number of clusters of individuals using the software STRUCTURE: a simulation study. Mol. Ecol. 14, 2611–2620. 10.1111/j.1365-294X.2005.02553.x 15969739

[B18] Excoffier L. Laval G. Schneider S. (2005). Arlequin (version 3.0): an integrated software package for population genetics data analysis. Evol. Bioinform. 1, 47–50. 10.1143/JJAP.34.L418 PMC2658868 19325852

[B19] Farris J. S. Kallersjo M. Kluge A. G. Bult C. (1994). Testing significance of incongruence. Cladistics 10, 315–319. 10.1111/j.1096-0031.1996.tb00196.x

[B20] Feng X. Y. Liu J. Gong X. (2016). Species delimitation of the* Cycas segmentifida* complex (Cycadaceae) resolved by phylogenetic and distance analyses of molecular data. Front. Plant Sci. 7, 134–145. 26913044 10.3389/fpls.2016.00134 PMC4753401

[B21] Feng X. Y. Wang Y. H. Gong X. (2014). Genetic diversity, genetic structure and demographic history of *Cycas simplicipinna* (Cycadaceae) assessed by DNA sequences and SSR markers. BMC Plant Biol. 14, 187–203. 10.1186/1471-2229-14-187 25016306 PMC4114127

[B22] Feng X. Y. Liu J. Chiang Y. C. Gong X. (2017). Investigating the genetic diversity, population differentiation and population dynamics of *Cycas segmentifida* (Cycadaceae) Endemic to Southwest China by multiple molecular Markers. Front. Plant Sci. 8, 839–871. 10.3389/fpls.2017.00839 28580005 PMC5437697

[B23] Fragniere Y. Betrisey S. Cardinaux L. Stoffel M. Kozlowski G. (2015). Fighting their last stand? A global analysis of the distribution and conservation status of gymnosperms. J. Biogeogr. 42, 809–820. 10.1111/jbi.12480

[B24] Fu G. A. (2004). A new species of genus Cycas from China. Bull. Bot. Res. 24, 387–388.

[B25] Fu Y. X. (1997). Statistical tests of neutrality of mutations against population growth, hitchhiking and background selection. Genet. 147, 915–925. 10.0000/PMID9335623 PMC1208208 9335623

[B26] Funk W. C. McKay J. K. Hohenlohe P. A. Allendorf F. W. (2012). Harnessing genomics for delineating conservation units. Trends Ecol. Evol. 27, 489–496. 10.1016/j.tree.2012.05.012 22727017 PMC4185076

[B27] Gong Y. Q. Gong X. (2016). Pollen-mediated gene flow promotes low nuclear genetic differentiation among populations of *Cycas debaoensis* (Cycadaceae). Tree Genet. Genomes 12, 93–108. 10.1007/s11295-016-1051-6

[B28] Gong Y. Zhan Q. Q. Khang S. N. (2015). The historical demography and genetic variation of the endangered *Cycas multipinnata* (Cycadaceae) in the Red River region, examined by chloroplast DNA sequences and microsatellite markers. PloS One, 10 (2). 10.1371/journal.pone.0117719 PMC4331093 25689828

[B29] Graur D. Li W. H. (2000). Fundamentals of molecular evolution. (Sunderland: Sinauer Associates).

[B30] Gregory T. Lopez-Gallego C. (2018). No more cycad extinctions: a line in the sand. Cycads 3, 4–5.

[B31] Griffith M. P. Calonje M. Meerow A. W. Tut F. Kramer A. T. Hird A. (2015). Can a botanic garden cycad collection capture the genetic diversity in a wild population? Intl. J. Plant Sci. 176, 1–10. 10.1086/678466

[B32] Griffith M. P. Calonje M. Meerow A. W. Francisco-Ortega J. Knowles L. Aguilar R. (2017). Will the same ex situ protocols give similar results for closely related species? Biodivers. Conserv. 26, 2951–2966. 10.1007/s10531-017-1400-2

[B33] Gutiérrez-Ortega J. S. Salinas-Rodriguez M. Martinez J. F. Molina-Freaner F. Perez-Farrera M. Vovides A. P. (2017). The phylogeography of the cycad genes Dioon (Zamiaceae) clarifies its Cenozoic expansion and diversification in the Mexican transition zone. Ann. Bot. 121, 535–548. 10.1093/aob/mcx165 PMC5838841 29293877

[B34] Hall J. A. Walter G. H. (2011). Does pollen aerodynamics correlate with pollination vector? Pollen settling velocity as a test for wind versus insect pollination among cycads (Gymnospermae: Cycadaceae: Zamiaceae). Biol. J. Linn. Soc 104, 75–92. 10.1111/j.10958312.2011.01695.x

[B35] Hall T. A. (1999). BioEdit: a user-friendly biological sequence alignment editor and analysis program for Windows 95/98/NT. Nucleic Acids Symp. Ser. 41, 95–98.

[B36] Harrison S. P. Yu G. Takahara H. Prentice I. C. (2001). Palaeovegetation - Diversity of temperate plants in east Asia. Nature 413, 129–130. 10.1038/35093166 11557970

[B37] Hewitt G. (2000). The genetic legacy of the Quaternary ice ages. Nature 405, 907–913. 10.1038/35016000 10879524

[B38] Hewitt G. M. (2004). Genetic consequences of climatic oscillations in the Quaternary. Philos. T. R. Soc B-Biol. Sci. 359, 183–195. 10.1098/rstb.2003.1388 PMC1693318 15101575

[B39] Huang Y. Guo X. Ho S. Y. W. Shi H. Li J. Li J. (2013a). Diversification and demography of the oriental garden lizard (Calotes versicolor) on Hainan island and the adjacent mainland. PloS One 8 (6), e64754. 10.1371/journal.pone.0064754 23840304 PMC3694074

[B40] Huang Y. F. Liao S. B. Chen Y. Luo S. X. Liu D. W. Cai G. (2013b). Population characteristics and conservation of *Cycas fairylakea*. For. Res. 26, 668–672. 10.1007/s11632-012-0208-0

[B41] Huang Y. Y. (2001). Systematic classification and evolution of Cycadaceae plants in China. (Beijing: China Meteorological Press).

[B42] James H. E. Forster P. I. Lamont R. W. Shapcott A. (2018). Conservation genetics and demographic analysis of the endangered cycad species *Cycas megacarpa* and the impacts of past habitat fragmentation. Aust. J. Bot. 66, 173–189. 10.1071/bt17192

[B43] Jensen R. J. (1989). NTSYS-PC - numerical taxonomy and multivariate-analysis system-version 1.40. Q. Rev. Biol. 64, 250–252. 10.1086/416356

[B44] Jian S. G. Liu N. Gao Z. Z. Wei Q. Xie Z. H. Wu M. (2005a). Biological characteristics of wild *Cycas fairylake* a population in Guangdong province. Acta Sci. Natural. Univ. Sunyatseni 44, 97–100. 10.1007/s11515-006-0058-z

[B45] Jian S. G. Wei Q. Gao Z. Xie Z. Lin S. Liu N. (2005b). Characteristics and conservation of wild populations of *Cycas fairylakea* newly found in Qujiang of Guangdong Province. Guihaia 25, 97–101. 10.1360/biodiv.050022

[B46] Jian S. G. Wu M. Liu N. (2005c). Genetic diversity of *Cycas changjiangensis* detected by allozyme analysis. Guihaia 25, 566–569. 10.1360/aps040037

[B47] Jian S. G. Zhong Y. Liu N. Gao Z. Z. Wei Q. Xie Z. H. (2006). Genetic variation in the endangered endemic species *Cycas fairylakea* (Cycadaceae) in China and implications for conservation. Biodivers. Conserv. 15, 1681–1694. 10.1007/s10531-004-5017-x

[B48] Jones D. L. (2002). Cycads of the world: ancient plant in today’s landscape (Washington: Smithsonian Institution Press).

[B49] Jump A. S. Penuelas J. (2005). Running to stand still: adaptation and the response of plants to rapid climate change. Ecol. Lett. 8, 1010–1020. 10.1111/j.1461-0248.2005.00796.x 34517682

[B50] Li L. Wang Z. F. Jian S. G. Zhu P. Zhang M. Ye W. H. (2009). Isolation and characterization of microsatellite loci in endangered *Cycas changjiangensis* (Cycadaceae). Conserv. Genet. 10, 793–795. 10.1007/s10592-008-9664-4

[B51] Librado P. Rozas J. (2009). DnaSP v5: a software for comprehensive analysis of DNA polymorphism data. Bioinformatics 25, 1451–1452. 10.1093/bioinformatics/btp187 19346325

[B52] Lin N. Deng T. Moore M. J. Sun Y. Huang X. Sun W. (2018). Phylogeography of Parasyncalathium souliei (Asteraceae) and its potential application in delimiting phylogeoregions in the Qinghai-Tibet Plateau (QTP)-Hengduan Mountains (HDM) Hotspot. Front. Genet. 9, 171. 10.3389/fgene.2018.00171 29868119 PMC5966570

[B53] Liu N. Qin G. (2004). Notes on some species of Cycas (Cycadaceae) from China. Proceedings of the Sixth International Conference on Cycad Biology, 2002, 1–4. Nong Nooch Tropical Garden, Bangkok, Thailand.

[B54] Liu J. Zhou W. Gong X. (2015). Species delimitation, genetic diversity and population historical dynamics of *Cycas diannanensis* (Cycadaceae) occurring sympatrically in the Red River region of China. Front. Plant Sci. 6, 696–701. 10.3389/fpls.2015.00696 26442013 PMC4562272

[B55] Liu J. Zhang S. Nagalingum N. Chiang Y. C. Lindstrom A. Gong X. (2018). Phylogeny of the gymnosperm genus *Cycas* L. (Cycadaceae) as inferred from plastid and nuclear loci based on a large-scale sampling: Evolutionary relationships and taxonomical implications. Mol. Phylogenet. Evol. 127, 87–97. 10.1016/j.ympev.2018.05.019 29783022

[B56] Liu N. (1998). A new species of the genus *Cycas* from Hainan island, China. Acta Phytotax. 36, 552–554. 10.7525/j.issn.1673-5102.2006.01.002

[B57] Lo´pez -Pujol J. Zhang F. M. Sun H. Q. Ying T. S. Ge S. (2011). Centres of plant endemism in China: places for survival or for speciation? J. Biogeogr. 38, 1267–1280. 10.1111/j.1365-2699.2011.02504.x

[B58] Lo´pez-Pujol J. Zhang F. M. Ge S. (2006). Plant biodiversity in China: richly varied, endangered and in need of conservation. Biodivers. Conserv. 15, 3983–4026. 10.1007/s10531-005-3015-2

[B59] Mantel N. (1967). Detection of disease clustering and a generalized regression approach. Cancer Res. 27, 209. 10.1007/s00253-002-1013-9 -& 6018555

[B60] Mekonnen A. Rueness E. K. Stenseth N. C. Fashing P. J. Bekele A. Hernandez-Aguilar R. A. (2018). Population genetic structure and evolutionary history of Bale monkeys (*Chlorocebus djamdjamensis*) in the southern Ethiopian Highlands. BMC Evol. Biol. 18, 106–121. 10.1186/s12862-018-1217-y 29986642 PMC6038355

[B61] Meerow A. W. Salas-Leiva M. Calonje J. Francisco-Ortega M. P. Griffith K. Nakamura F. (2018) Contrasting demographic history and population structure of Zamia (Cycadales: Zamiaceae) on six islands of the Greater Antilles suggests a model for population diversification in the Caribbean clade of the genus. International J. Plant Sci. 179, 730–757.

[B62] Mittermeler R. A. (2017). Hotspots Revisited - Earth's biologically richest and most endangered terrestrial ecoregions. (2), 237–238.

[B63] Mo P. Q. Huang Y. Y. Zhong X. Q. Liu G. L. Li Z. W. Nong B. X. (2008). Establishment of *Cycas micholitzii* ISSR-PCR optimal conditions with orthogonal optimization method. Bull. Bot. Res. 28, 304–309. 10.7525/j.issn.1673-5102.2008.03.013

[B64] Montalvo A. M. Williams S. L. Rice K. J. Buchmann S. L. Cory C. Handel S. N. (1997). Restoration biology: A population biology perspective. Restor. Ecol. 5, 277–290. 10.1046/j.1526-100X.1997.00542.x

[B65] Myers N. Mittermeier R. A. Mittermeier C. G. da Fonseca G. A. B. Kent J. (2000). Biodiversity hotspots for conservation priorities. Nature 403, 853–858. 10.1038/35002501 10706275

[B66] Nagalingum N. S. Marshall C. R. Quental T. B. Rai H. S. Little D. P. Mathews S. (2011). Recent synchronous radiation of a living fossil. Science 334, 796–799. 10.1126/science.1209926 22021670

[B67] Nayak S. Naik P. K. Acharya L. Mukherjee A. K. Panda P. C. Das P. (2005). Assessment of genetic diversity among 16 promising cultivars of ginger using cytological and molecular markers. Zeitschrift Fur Naturforschung Section C-A. J. Biosci. 60, 485–492. 10.1515/znc-2005-5-618 16047412

[B68] Nong B. X. Huang Y. Y. Liu C. (2011). Genetic relationships analysis in some species of *Cycas* in China by RAPD markers. Guihaia 31, 167–174. 10.3724/SP.J.1011.2011.00093

[B69] Nybom H. (2004). Comparison of different nuclear DNA markers for estimating intraspecific genetic diversity in plants. Mol. Ecol. 13, 1143–1155. 10.1111/j.1365-294X.2004.02141.x 15078452

[B70] Nylander J. A. (2004). MrModeltest v2.2. Program Distributed by the author. Bioinformatics. 24 581–583. 10.4236/bio.2004.48074

[B71] Pauls S. U. Nowak C. Balint M. Pfenninger M. (2013). The impact of global climate change on genetic diversity within populations and species. Mol. Ecol. 22, 925–946. 10.1111/mec.12152 23279006

[B72] Peakall R. Smouse P. E. (2012). GenAlEx 6.5: genetic analysis in Excel. Population genetic software for teaching and research-an update. Bioinformatics 28, 2537–2539. 10.1093/bioinformatics/bts460 22820204 PMC3463245

[B73] Petit R. J. Mousadik A. Pons O. (1998). Identifying populations for conservation on the basis of genetic markers. Conserv. Biol. 12, 844–855. 10.1046/j.1523-1739.1998.96489.x

[B74] Petit R. J. Duminil J. Fineschi S. Hampe A. Salvini D. Vendramin G. G. (2005). Comparative organization of chloroplast, mitochondrial and nuclear diversity in plant populations. Mol. Ecol. 14, 689–701. 10.1111/j.1365-294X.2004.02410.x 15723661

[B75] Piry S. Luikart G. Cornuet J. M. (1999). BOTTLENECK: a computer program for detecting recent reductions in the effective population size using allele frequency data. J. Hered. 90, 502–503. 10.1093/jhered/90.4.502

[B76] Pons O. Petit R. J. (1996). Measuring and testing genetic differentiation with ordered versus unordered alleles. Genet. 144, 1237–1245. 10.1016/S1050-3862(96)00162-3 PMC1207615 8913764

[B77] Pritchard J. K. Stephens M. Donnelly P. (2000). Inference of population structure using multilocus genotype data. Genetics 155 (2), 945–959. 10835412 10.1093/genetics/155.2.945 PMC1461096

[B78] Rambaut A. Suchard M. Xie D. Drummond A. (2014). Tracer v1.6, Oxford: University of Oxford Available at: http://tree.bio.ed.ac.uk/software/tracer/

[B79] Reisch C. Bernhardt-Roemermann M. (2014). The impact of study design and life history traits on genetic variation of plants determined with AFLPs. Plant Ecol. 215, 1493–1511. 10.1007/s11258-014-0409-9

[B80] Reisch C. Schmidkonz S. Meier K. Schoepplein Q. Meyer C. Hums C. (2017). Genetic diversity of calcareous grassland plant species depends on historical landscape configuration. BMC Ecol. 17, 19–32. 10.1186/s12898-017-0129-9 28438203 PMC5404287

[B81] Ronquist F. Teslenko M. van der Mark P. Ayres D. L. Darling A. Hohna S. (2012). MrBayes 3.2: efficient Bayesian phylogenetic inference and model choice across a large model space. Syst. Biol. 61, 539–542. 10.1093/sysbio/sys029 22357727 PMC3329765

[B82] Rosenberg N. A. (2004). DISTRUCT: a program for the graphical display of population structure. Mol. Ecol. Notes 4, 137–138. 10.1046/j.1471-8286.2003.00566.x

[B83] Rousset F. (2008). GENEPOP ‘ 007: a complete re-implementation of the GENEPOP software for Windows and Linux. Mol. Ecol. Resour. 8, 103–106. 10.1111/j.1471-8286.2007.01931.x 21585727

[B84] Salas-Leiva D. E. Meerow A. W. Calonje M. Griffith M. P. Francisco-Ortega J. Nakamura K. (2013). Phylogeny of the cycads based on multiple single-copy nuclear genes: congruence of concatenated parsimony, likelihood and species tree inference methods. Ann. Bot. 112, 1263–1278. 10.1093/aob/mct192 23997230 PMC3806525

[B85] Santiago-Jimnez Q. J. Martnez-Dominguez L. Nicolalde-Morejn F. (2019). Two new Mexican species of *Pharaxonotha Reitter*, 1875 (Coleoptera: Erotylidae) from *Ceratozamia tenuis* (Cycadales: Zamiaceae). Dugesiana 26, 15–25. 10.32870/dugesiana.v26i1.7054

[B86] Schneider D. Wink M. Sporer F. Lounibos P. (2002). Cycads: their evolution, toxins, herbivores and insect pollinators. Sci. Nat. 89, 281–294. 10.1007/s00114-002-0330-2 12216856

[B87] Shi Y. F. Cui Z. J. Su Z. (2006). The Quaternary Glaciations and Environmental Variations in China (Shijiazhuang: Hebei Science and Technology Press).

[B88] Swindell S. R. Plasterer T. N. (1997). SEQMAN. Contig assembly. Methods Mol. Bio. (Clifton N.J.) 70, 75–89. 10.1385/0-89603-358-9:75 9089604

[B89] Swofford D. L. (2002). Phylogeny analysis using parsimony (* and other methods). Version 4 (Sunderland, MA: Sinauer Associates).

[B90] Taberlet P. Fumagalli L. Wust-Saucy A. G. Cosson J. F. (1998). Comparative phylogeography and postglacial colonization routes in Europe. Mol. Ecol. 7, 453–464. 10.1046/j.1365-294x.1998.00289.x 9628000

[B91] Tajima F. (1989). Statistical-method for testing the neutral mutation hypothesis by DNA polymorphism. Genet. 123, 585–595. 10.0000/PMID2513255 PMC1203831 2513255

[B92] Tang W. Liu N. Lindström A. J. Tang L. Calonje M. A. (2018a). Cycas debaoensis conservation project–a village-based approach. Mem. N. Y. Bot. Gard. 117, 95–105. 10.21135/893275389.009

[B93] Tang W. Skelley P. Pérez-Farrera M. A. (2018b). Ceratophila, a new genus of erotylid beetles (Erotylidae: Pharaxono-thinae) inhabiting male cones of the cycad Ceratozamia (Cycadales: Zamia-ceae). Zootaxa. 4508, 151–178. 10.3390/d10020038 30485971

[B94] Van O. C. Joyce D. A. Cummings S. M. Blais J. Barson N. J. Ramnarine I. W. (2010). Balancing selection, random genetic drift, and genetic variation at the major histocompatibility complex in two wild populations of guppies (Poecilia reticulata). Evolution 60 (12), 2562–2574. 10.1554/06-286.1 17263117

[B95] Viechtbauer W. (2010). Conducting meta-analyses in R with the metafor package. J. Stat. Software 36, 1–48.

[B96] Vovides A. P. Galicia S. (2016). G-fibers and florin ring-like structures in Dioon (Zamiaceae). Bot. Sci. 94, 263–268.

[B97] Wang J. Ai B. Kong H. Kang M. (2017). Speciation history of a species complex of *Primulina eburnea* (Gesneriaceae) from limestone karsts of southern China, a biodiversity hot spot. Evol. Appl. 10, 919–934. 10.1111/eva.12495 29151883 PMC5680421

[B98] Wang D. Y. (2000). Morphological structure, system classification and evolutionary research of Cycadaceae (Nanjing: Nanjing Forestry University).

[B99] Wei Z. Guan M. M. Gong X. (2015). *Cycas chenii* (Cycadaceae), a new species from China, and its phylogenetic position. J. Syst. Evol. 53, 489–498. 10.1111/jse.12153

[B100] Wolfe K. H. Li W. H. Sharp P. M. (1987). Rates of nucleotide substitution vary greatly among plant mitochondrial, chloroplast, and nuclear DNA. Proc. Natl. Acad. Sci. U. S. A. 84, 9054–9058. 10.1073/pnas.84.24.9054 3480529 PMC299690

[B101] Wright S. (1931). Evolution in Mendelian populations. Genetics 16 (2), 97. 17246615 10.1093/genetics/16.2.97 PMC1201091

[B102] Wright S. (1978). Evolution and the genetics of populations: a treatise in four volumes. Variability within and among natural populations. (Chicago: University of Chicago Press).

[B103] Wu Y. Huang J. Zhang M. Luo S. Zhang Y. Lei F. (2012). Genetic divergence and population demography of the Hainan endemic Black-throated Laughingthrush (Ayes: Timaliidae, Garrulax chinensis monachus) and adjacent mainland subspecies. Mol. Phylogenet. Evol. 65, 482–489. 10.1016/j.ympev.2012.07.005 22820021

[B104] Xiao L. Q. Moeller M. (2015). Nuclear ribosomal its functional paralogs resolve the phylogenetic relationships of a late-miocene radiation cycad cycas (Cycadaceae). PloS One 10 (1), e0117971. 10.1371/journal.pone.0117971 25635842 PMC4311995

[B105] Xiao S. Y. Ji Y. H. Liu J. Gong X. (2018). Genetic characterization of *Cycas panzhihuaensis* (Cycadaceae): crisis lurks behind a seemingly bright prospect. PeerJ Preprints. 10.7287/peerj.preprints.27265v1

[B106] Yang S. L. Meerow A. W. (1996). The *Cycas pectinata* (Cycadaceae) Complex: Genetic Structure and Gene Flow. Int. J. Plant Sci. 157, 468–483. 10.1086/297364

[B107] Yang Y. Q. Huang B. H. Yu Z. X. Liao P. C. (2015). Inferences of demographic history and fine-scale landscape genetics in *Cycas panzhihuaensis* and implications for its conservation. Tree Genet. Genomes 11, 78–93. 10.1007/s11295-015-0894-6

[B108] Yang Y. Duke N. C. Peng F. Li J. Yang S. Zhong C. (2016). Ancient geographical barriers drive differentiation among *Sonneratia caseolaris* populations and recent divergence from. S. Lanceolata. Front. Plant Sci. 7, 1618–1632. 10.3389/fpls.2016.01618 27833634 PMC5080369

[B109] Zhan Q. Q. Wang J. F. Gong X. Peng H. (2011). Patterns of chloroplast DNA variation in *Cycas debaoensis* (Cycadaceae): conservation implications. Conserv. Genet. 12, 959–970. 10.1007/s10592-011-0198-9

[B110] Zhang M. Wang Z. F. Jian S. G. Ye W. H. Cao H. L. Zhu P. (2009). Isolation and characterization of microsatellite markers for *Cycas hainanensis* C. J. Chen (Cycadaceae). Conserv. Genet. 10, 1175–1176. 10.1007/s10592-008-9737-4

[B111] Zhang H. J. Feng T. Landis J. B. Deng T. Zhang X. Meng A. P. (2019). Molecular phylogeography and ecological niche modeling of sibbaldia procumbens s.l. (Rosaceae). Front. Genet. 10, 201. 10.3389/fgene.2019.00201 30918513 PMC6424895

[B112] Zhao Y. J. Gong X. (2015). Genetic divergence and phylogeographic history of two closely related species (*Leucomeris decora* and *Nouelia insignis*) across the ‘Tanaka Line’ in Southwest China. BMC Evol. Biol. 15, 134–147. 10.1186/s12862-015-0374-5 26153437 PMC4495643

[B113] Zhao S. Zheng P. P. Dong S. S. (2013). Whole-genome sequencing of giant pandas provides insights into demographic history and local adaptation. Nature Genet. 45, 67–71. 23242367 10.1038/ng.2494

[B114] Zhao T. H. Wang L. R. Yuan J. Y. (2007). Origin and time of Qiongzhou strait. Marine Geol. Quater. Geol. 27, 33–40. 10.16562/j.cnki.0256-1492

[B115] Zheng Y. Liu J. Gong X. (2016). Tectonic and climatic impacts on the biota within the red river fault, evidence from phylogeography of *Cycas dolichophylla* (Cycadaceae). Sci. Rep. 6, 33540. 10.1038/srep33540 27629063 PMC5024324

[B116] Zheng Y. Liu J. Feng X. Y. Gong X. (2017). The distribution, diversity, and conservation status of *Cycas* in China. Ecol. Evol. 7, 3212–3224. 10.1002/ece3.2910 28480020 PMC5415521

