# Supplementary material for: Population Differentiation and Demographic History of the Cycas taiwaniana Complex (Cycadaceae) Endemic to South China as Indicated by DNA Sequences and Microsatellite Markers
Source: Front Genet. 2019 Dec 23;10:1238. doi: 10.3389/fgene.2019.01238 (PMC6935862; doi:10.3389/fgene.2019.01238)
Supplement: Supplementary file 1 [file DataSheet_1.pdf]

frontiers

Supplementary Material

Population differentiation and demographic history of the *Cycas taiwaniana* complex (Cycadaceae) endemic to South China as indicated by DNA sequences and microsatellite markers

Xin-Hui Wang[1,2], Jie Li[1], Li-Min Zhang[1], Zi-Wen He[3], Qi-Ming Mei[1], Xun Gong[4] and Shu-Guang Jian[1]*

***Correspondence:** Shuguang Jian jiansg@scbg.ac.cn

1 Supplementary Figures and Tables

1.1 Supplementary Figures

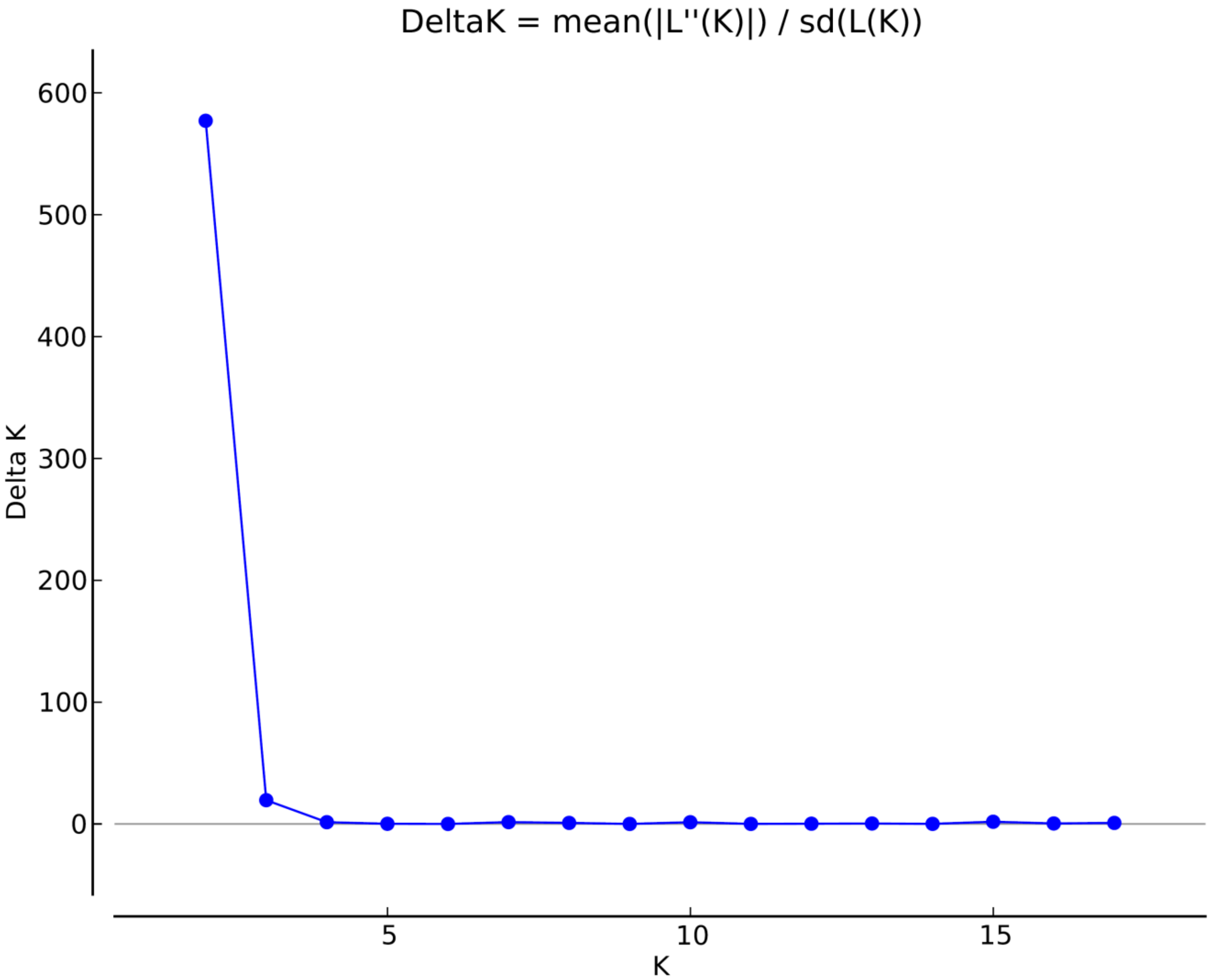

Supplementary Figure 1. ΔK plot based on SSR.

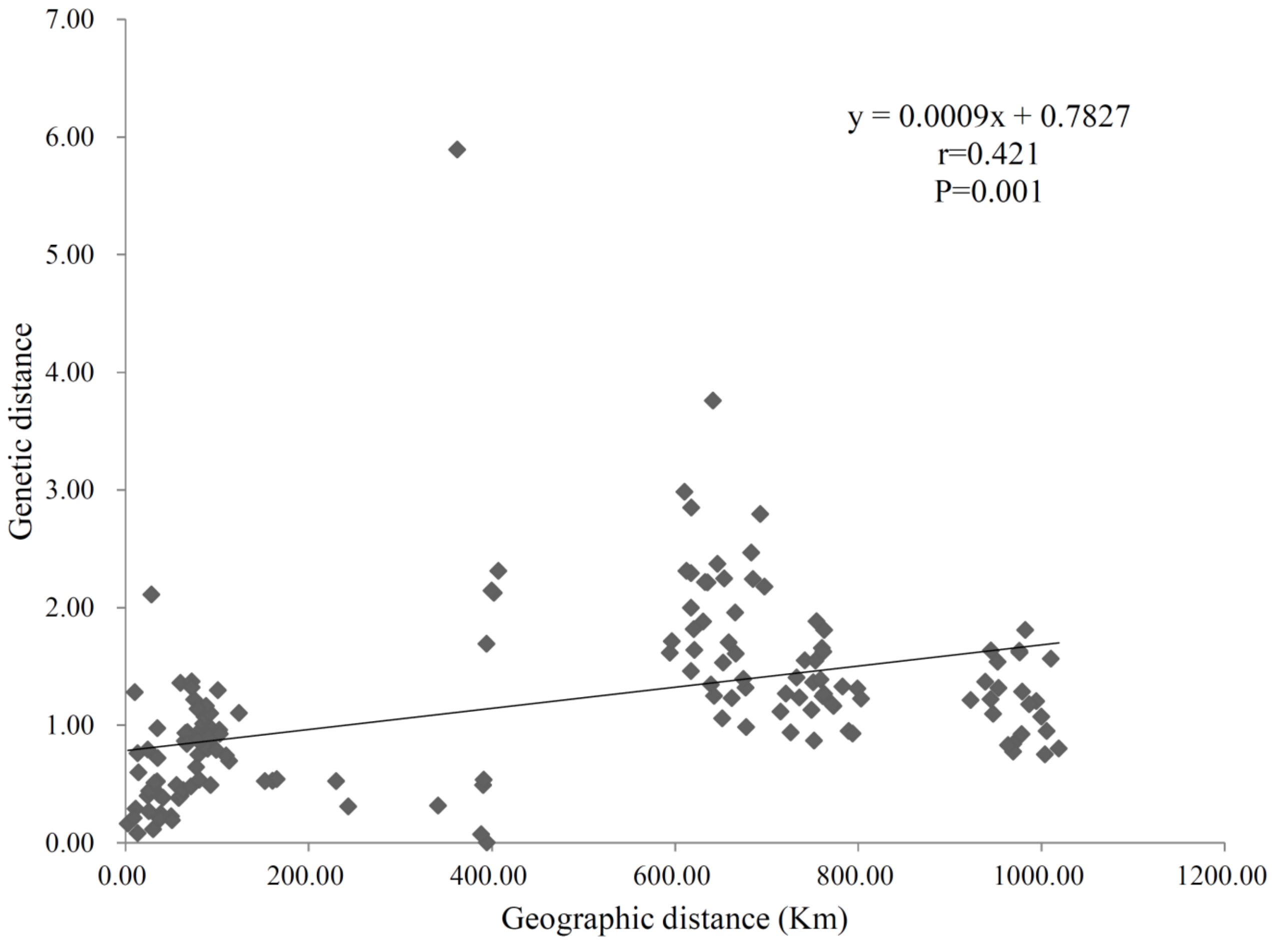

Supplementary Figure 2. Scatter plot of geographic distance against genetic distance for 18 populations of the *C. taiwaniana* complex.

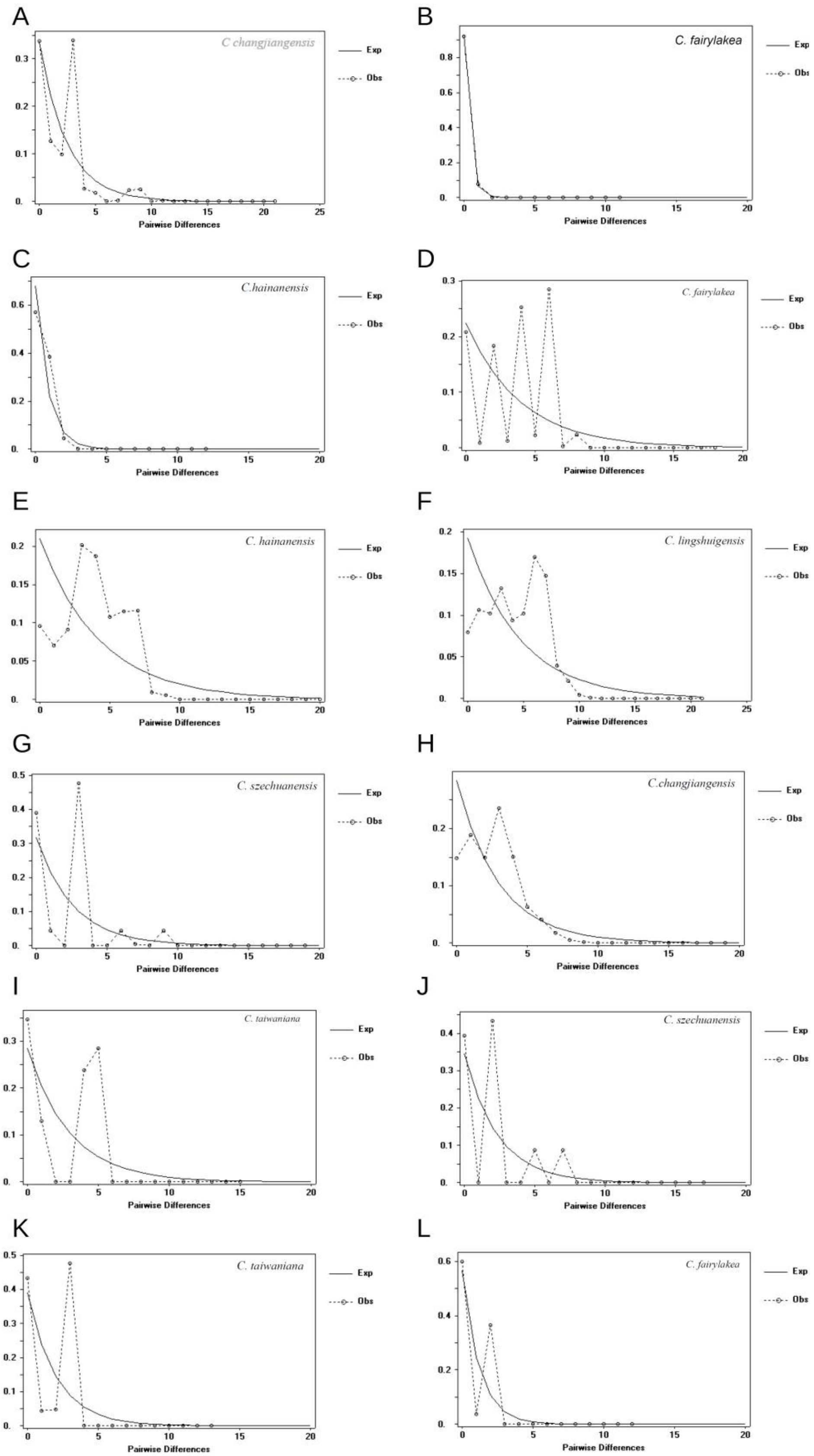
A
C changjiangensis
Exp
Obs
Pairwise Differences
B
C. fairylakea
Exp
Obs
Pairwise Differences
C
C.hainanensis
Exp
Obs
Pairwise Differences
D
C. fairylakea
Exp
Obs
Pairwise Differences
E
C. hainanensis
Exp
Obs
Pairwise Differences
F
C. lingshuigensis
Exp
Obs
Pairwise Differences
G
C. szechuanensis
Exp
Obs
Pairwise Differences
H
C.changjiangensis
Exp
Obs
Pairwise Differences
I
C. taiwaniana
Exp
Obs
Pairwise Differences
J
C. szechuanensis
Exp
Obs
Pairwise Differences
K
C. taiwaniana
Exp
Obs
Pairwise Differences
L
C. fairylakea
Exp
Obs
Pairwise Differences

M

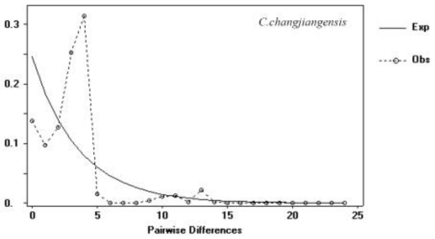
C.changjiangensis
Exp
Obs
Pairwise Differences
0.3
0.2
0.1
0.
0
5
10
15
20
25

N

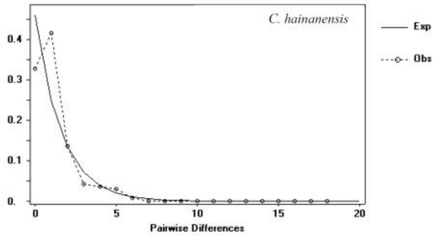
C. hainanensis
Exp
Obs
Pairwise Differences
0.4
0.3
0.2
0.1
0.
0
5
10
15
20

O

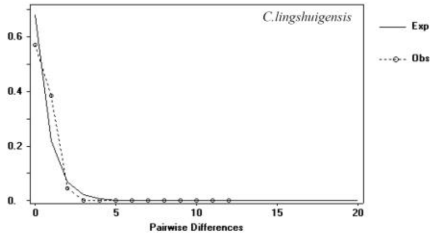
C.lingshuigensis
Exp
Obs
Pairwise Differences
0.6
0.4
0.2
0.
0
5
10
15
20

Supplementary Figure 3. Mismatch distribution analysis plots for six taxa in the *C. taiwaniana* complex based on cpDNA (**A**, **B** and **C**), EX (**D**—**I**) and FJ (**J**—**O**). The solid lines indicate expected distribution, whereas dotted lines denote observed values.

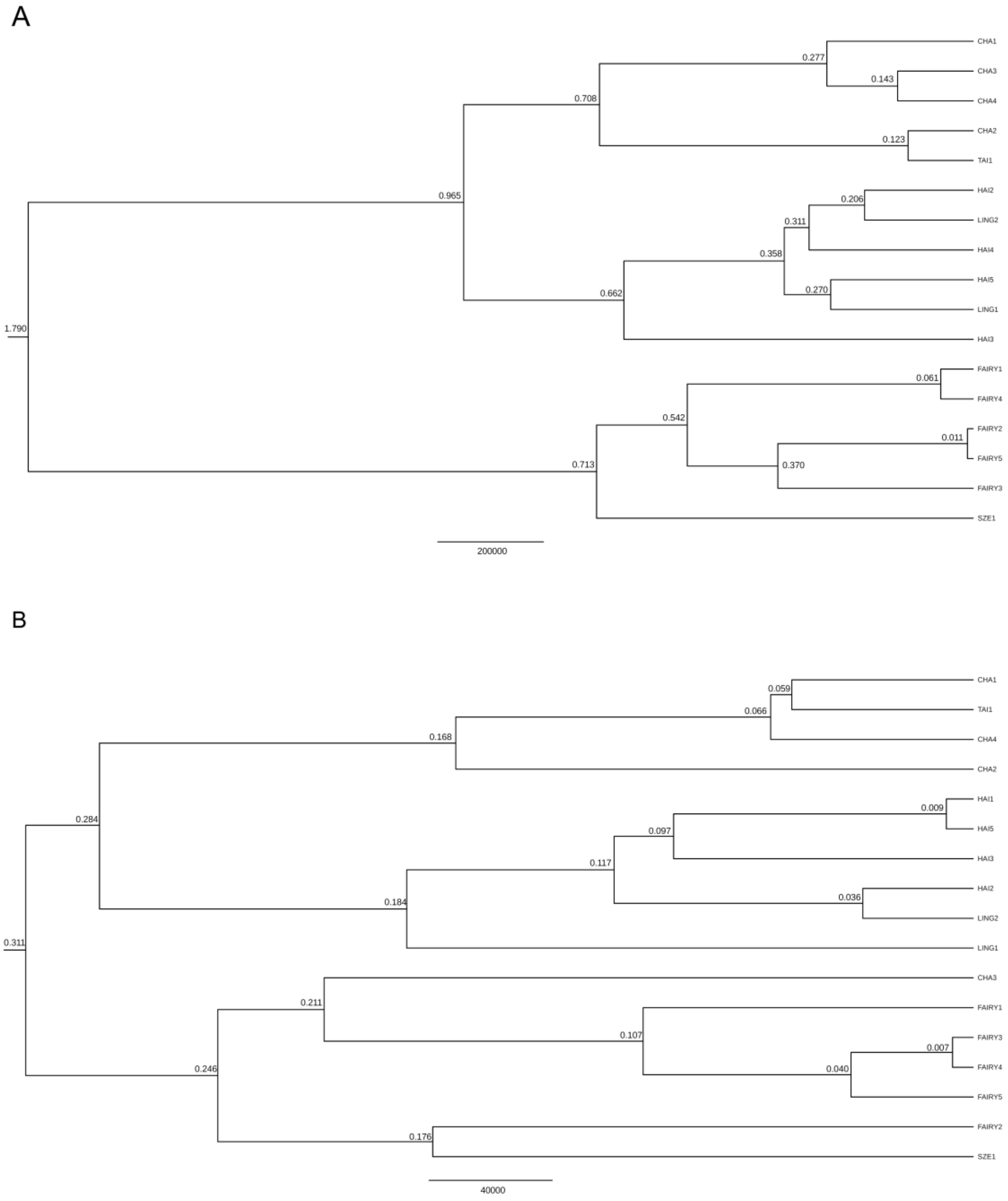

Supplementary Figure 4. BEAST-derived chronogram for combined cpDNA **(A)** and SCNGs **(B)** of the *C. taiwaniana* complex. The value at each node indicates the estimated divergent time (MYA).

1.2 Supplementary Tables

Supplementary Table 1. Information of primers for the cpDNA and SCNG loci used in this study.

Locus	Primer sequences (5'-3')	Length (bp)	Tm(°C)	Reference
trnS-trnG	GCCGCTTTAGTCCACTCAGC<br>GAACGAATCACACTTTTACCAC	877	53	Hamilton, 1999
atpB-rbcL	ACATCKARTACKGGACCAATAA<br>AACACCAGCTTTRAATCCAA	717	55	Chiang et al., 1998
EX929503	TCACCAGATTTGAAGATGGC<br>CCCCTCTTCAAATCGAGGTTG	1069	55	Unpublished
FJ393265	CCAGTCTCCCAGTATCATGG<br>GCTGCATGATATTTCCAACC	1106	58	Unpublished

Supplementary Table 2. Information of primers for microsatellites (SSR)、the chloroplast DNA (cpDNA) fragments and single-copy nuclear genes (SCNGs) loci used in this study.

Locus	Primer sequences (5'-3')	Repeat motif	Tm(°C)	Reference
Cha02	CGAGGAACATCAAGGCTATG<br>CCTAGCTTTTGGGAATTAGAC	$(CT)_{21}$	60	Zhang et al., 2009
Cha03	TCAAATAATAGTGCCTAGAACC<br>AAGAGGCCATCTTGTCTC	$(AC)_{15}$	53	Zhang et al., 2009
Cha05	GTCTGCTAACATCTATAAA<br>GATGAGCTAAGAGTCATAGTA	$(CT)_{19}$	53	Zhang et al., 2009
Cha08	CAGGGACCATTGTTTCTAAGG<br>ACTTATACATAGGGCTCTAAT	$(AG)_{10}$	57	Zhang et al., 2009
HL02	GGGGTTCATATCACATAAC<br>CTATAAAGAATCATCGTTCTC	$(GT)_{17}(GA)_{11}$	50	Li et al., 2009
HL08	CAAAACATTCCTTGCCCTGT<br>GGGAGCCTGTTGAAGAGAGCTA	$(TTC)_{12}$	62	Li et al., 2009

Supplementary Table 3. Genetic diversity and the composition of haplotypes estimated from cpDNA and SCNGs in the *C. taiwaniana* complex.

Species	Pop. code	cpDNA			EX			FJ		
		Haplotypes (No.)	Hd	Pi $\times 10^3$	Haplotypes (No.)	Hd	Pi $\times 10^3$	Haplotypes (No.)	Hd	Pi $\times 10^3$
C.lingshuigensis	LING1	H1(10)	0	0	H1(2)H6(1)H7(1) H11(2)H20(3)H22 (2)H25(5)H27(1) H28(1)H29(1)H30 (1)H31(1)H32(1)	0.93	4.5	H4(11)H8(9)H20 (1)H21(1)	0.61	0.9
	LING2	H1(9)	0	0	H1(1)H7(3)H11(6) H22(1)H24(3)H25 (3)H29(1)H35(1) H36(1)H37(1)H38 (1)	0.91	4.3	H4(21)H8(1)	0.09	0.1
	Total		0	0		0.93	4.5		0.43	0.6
C. hainanensis	HAI1	H1(10)	0	0	H2(2)E9(1)H11(6) H20(2)H22(4)H23 (1)H24(1)H25(2) H26(1)	0.87	4.4	H4(7)H8(6)H15(3)H16(1)H17(1) H18(1) H19(1)	0.77	2.1
	HAI2	H3(10)	0	0	H1(3)H2(1)H25(6) H32(7)H46(1)H47 (1)H48(1)	0.78	3.0	H1(2)H4(1)H8(8) H15(6)H24(1) H25(1)H26(1)	0.77	2.4
	HAI3	H1(10)	0	0	H1(3)H2(5)H9(1) H25(2)H26(2)H32 (3)H48(1)H49(1) H50(1) H51(1)	0.90	3.6	H4(15)H8(4) H15(1)	0.42	0.6
	HAI4	H1(9)	0	0	H2(2)H7(5)H25(7) H34(3)H48(3)	0.80	3.5	H8(15)H15(5)	0.42	0.5
	HAI5	H1(10)	0	0	H2(1)H7(2)H11(3) H22(4)H25(6)H33 (1) H34(1)	0.84	4.2	H4(13)H8(5)	0.43	0.5
	Total		0.39	0.3		0.90	4.0		0.67	1.5

C. taiwaniana	TAI1	H1(10)	0	0	H1(5)H2(6)H23(6) H56(5)	0.65	2.7	H1(10)H4(11) H29(1)	0.57	2.0
C. fairylakea	FAIRY1	H2(11)	0	0	H39(15)H40(7)	0.46	2.9	H11(2)H22(4) H23(16)	0.45	0.9
	FAIRY2	H2(10)	0	0	H7(9)H11(1)H41(9)H42(1)	0.62	2.4	H22(10)H23(10)	0.53	1.3
	FAIRY3	H2(10)	0	0	H43(10)H44(1) H45(9)	0.57	1.2	H22(20)	0	0
	FAIRY4	H2(10)	0	0	H43(11)H45(11)	0.52	1.1	H22(22)	0	0
	FAIRY5	H2(8)H9(2)	0.36	0.2	H43(11)H45(11)	0.52	1.1	H22(22)	0	0
	Total		0.08	0.1		0.79	3.7		0.40	1.0
C.changjiangensis	CHA1	H1(1)H4(3)H5(5)	0.64	0.7	H1(12)H2(1)H3(1) H4(1)H5(1)	0.45	1.1	H1(1)H2(6)H3(7) H4(1)H5(1)	0.70	2.2
	CHA2	H5(8)H6(1)	0.22	1.1	H1(8)H2(4)H4(1) H6(1)H7(1)H8(1) H9(2)	0.76	2.0	H1(7)H4(2)H6(1) H7(1)H8(4)H9(1) H10(1)H11(1)	0.80	3.2
	CHA3	H7(9)H8(1)	0.20	0.5	H1(1)H7(2)H10(1) H11(3)H12(1) H13(1)H14(2)H15 (4)H16(1)H17(1) H18(1)	0.92	3.5	H2(1)H4(11)H8(1)H9(1)H10(1)H 11(1)H12(1)H13(1)	0.64	3.8
	CHA4	H4(1)H7(9)	0.20	0.1	H1(4)H2(7)H6(4) H11(2)H19(1) H20(1)H21(1)	0.82	3.3	H1(7)H2(6)H4(2) H8(4)H14(1)	0.77	3.4
	Total		0.66	1.3		0.85	2.7		0.86	3.9
C. szechuanensis	SZE1	H2(10)	0	0	H7(9)H52(1)H53 (1)H54(10) H55(1)	0.61	2.3	H22(9)H23(11) H27(1)H28(1)	0.61	2.4
Total	18	9	0.73	3.2	56	0.94	4.8	29	0.83	2.8

No, Number of individuals; Hd, Haplotype diversity; Pi, Nucleotide diversity

Supplementary Table 4. Genetic diversity of the six microsatellite loci employed in the *C. taiwaniana* complex. Primer codes are explained in Table 2.

Locus	N_A	A_E	*I*	H_o	H_e	F_{IS}	F_{ST}
Cha02	21	6.934	1.142	0.523	0.489	-0.068	0.392
Cha03	17	5.469	1.055	0.577	0.568	-0.016	0.292
Cha05	16	4.658	1.046	0.587	0.519	-0.131	0.381
Cha08	24	6.399	1.218	0.780	0.636	-0.227	0.270
HL02	19	7.948	1.177	0.426	0.500	0.149	0.408
HL08	20	6.238	1.280	0.597	0.566	-0.055	0.362
Mean	19.5	6.473	1.153	0.582	0.546	-0.058	0.351

$N_{A,}$ Number of alleles; $A_{E,}$ Number of effective alleles; *I*, Shannon's diversity index; $H_{o,}$ Observed heterozygosity; $H_{e,}$ Expected heterozygosity; F, Fixation index; PPL, Percentage of polymorphic loci

Supplementary Table 5. Genetic diversity parameters of the six species.

Species	N_A	A_E	I	H_o	H_e	F	PPL (%)
C.lingshuigensis	11.500	5.948	1.986	0.753	0.810	0.073	100.00
C. hainanensis	12.677	5.682	1.935	0.658	0.795	0.174	100.00
C. taiwaniana	1.833	1.833	0.578	0.833	0.417	-1.000	83.33
C. fairylakea	3.167	2.027	0.748	0.453	0.439	-0.001	100.00
C.changjiangensis	14.333	6.545	2.139	0.659	0.830	-0.220	100.00
C. szechuanensis	2.667	1.762	0.609	0.563	0.376	0.212	100.00
Mean	7.694	3.966	1.332	0.653	0.611	-0.102	97.22

$N_{A,}$ Number of alleles; $A_{E,}$ Number of effective alleles; *I*, Shannon's diversity index; $H_{o,}$ Observed heterozygosity; $H_{e,}$ Expected heterozygosity; F, Fixation index; PPL, Percentage of polymorphic loci

Supplementary Table 6. Gene flows between each pair of the *C. taiwaniana* complex populations.

Pop.	LING1	LING2	HAI1	HAI2	HAI3	HAI4	HAI5	TAI1	FAIRY1	FAIRY2	FAIRY3	FAIRY4	FAIRY5	SZE1	CHA1	CHA2	CHA3	CHA4
LING1	0.000																	
LING2	25.711	0.000																
HAI1	7.783	11.677	0.000															
HAI2	3.015	2.248	2.928	0.000														
HAI3	3.201	4.590	2.598	2.154	0.000													
HAI4	4.330	5.368	3.115	1.710	2.072	0.000												
HAI5	6.081	8.928	3.933	2.088	2.095	16.176	0.000											
TAI1	0.755	0.742	0.693	0.638	0.583	0.689	0.746	0.000										
FAIRY1	0.382	0.341	0.301	0.352	0.382	0.305	0.321	0.141	0.000									
FAIRY2	0.368	0.374	0.272	0.266	0.312	0.333	0.310	0.117	0.178	0.000								
FAIRY3	0.410	0.397	0.352	0.398	0.419	0.369	0.391	0.155	0.466	0.217	0.000							
FAIRY4	0.380	0.407	0.324	0.369	0.424	0.385	0.398	0.138	0.229	0.163	1.132	0.000						
FAIRY5	0.494	0.459	0.429	0.468	0.495	0.432	0.467	0.168	0.476	0.207		1.286	0.000					
SZE1	0.482	0.415	0.427	0.474	0.426	0.387	0.405	0.179	0.295	0.140	0.381	0.317	0.410	0.000				
CHA1	1.276	0.979	1.172	1.374	0.961	0.959	1.026	0.359	0.357	0.174	0.308	0.247	0.357	0.360	0.000			
CHA2	1.524	1.156	1.293	1.111	1.083	1.018	0.975	0.576	0.285	0.229	0.304	0.259	0.351	0.345	1.059	0.000		
CHA3	2.173	1.540	1.711	1.664	1.379	1.195	1.298	0.586	0.294	0.252	0.354	0.312	0.415	0.377	0.797	1.461	0.000	
CHA4	1.701	1.274	1.130	0.995	0.979	0.972	0.936	0.438	0.285	0.310	0.326	0.294	0.379	0.325	0.881	1.363	1.344	0.000

Supplementary Table 7. The P-value of Hardy-Weinberg equilibrium test for 18 populations of the *C. taiwaniana* complex.

Population	Cha02	Cha03	Cha05	Cha08	HL02	HL08	All loci (0.01)
LING1	0.655^{ns}	0.865^{ns}	0.581^{ns}	0.524^{ns}	0.716^{ns}	0.063^{ns}	0.335^{ns}
LING2	0.144^{ns}	0.994^{ns}	0.997^{ns}	0.005**	0.001***	0.690^{ns}	0.016^{ns}
HAI1	0.362^{ns}	0.894^{ns}	0.804^{ns}	0.749^{ns}	0.654^{ns}	0.774^{ns}	0.037^{ns}
HAI2	0.000***	0.085^{ns}	0.000***	0.000***	0.009**	0.294^{ns}	0.000***
HAI3	0.896^{ns}	0.621^{ns}	0.054^{ns}	0.964^{ns}	0.126^{ns}	0.866^{ns}	0.194^{ns}
HAI4	0.165^{ns}	0.438^{ns}	0.608^{ns}	0.434^{ns}	0.000***	0.467^{ns}	0.000***
HAI5	0.000***	0.453^{ns}	0.050^{ns}	0.977^{ns}	0.430^{ns}	0.014*	0.000***
TAI1	-	0.001***	0.001***	0.001***	0.001***	0.001***	0.000***
FAIRY1	0.915^{ns}	0.425^{ns}	0.389^{ns}	0.232^{ns}	-	0.691^{ns}	0.636^{ns}
FAIRY2	-	-	0.000***	0.000***	-	-	0.000***
FAIRY3	-	-	0.005**	0.000***	0.000***	0.000***	0.000***
FAIRY4	-	-	0.814ns	0.000***	0.000***	0.000***	0.000***
FAIRY5	-	-	0.022*	0.001***	0.001***	0.001***	0.000***
CHA1	0.161^{ns}	0.837^{ns}	0.000***	0.198^{ns}	0.009**	0.061^{ns}	0.000***
CHA2	0.358^{ns}	0.491^{ns}	0.000***	0.733^{ns}	0.456^{ns}	0.001***	0.000***
CHA3	0.158^{ns}	0.225^{ns}	0.051^{ns}	0.052^{ns}	0.000***	0.966^{ns}	0.020^{ns}
CHA4	0.000***	0.796^{ns}	0.000***	0.000***	0.217^{ns}	0.074^{ns}	0.000***
SZE1	0.897^{ns}	0.000***	0.001**	0.000***	0.317^{ns}	0.000***	0.000***
All pop.	0.000***	0.859^{ns}	0.000***	0.100^{ns}	0.006**	0.869^{ns}	0.000***

-, Monomorphic; ns, non-significance; *P<0.05; **P<0.01; ***P<0.001

Supplementary Table 8. Neutrality tests and Mismatch distribution analysis of species across the *C. taiwaniana* complex based on combined cpDNA and two single-copy nuclear genes.

Marker	species	Fu and Li's *D**	Fu and Li's *F**	Tajima's *D*	SSD	Raggedness index
cpDNA	*C.changjiangensis*	0.5662	0.7756	0.9415	0.0171	0.2004
	C. hainanensis	-3.2305*	-3.0005*	-1.1226	0.0619	0.2008
	C. fairylakea	0.5415	0.1542	-0.8744	0.0061	0.7221
EX	*C.lingshuigensis*	0.60411	0.84848	0.95000	0.0195	0.0205
	C. hainanensis	-0.06567	0.34120	0.93127	0.0214	0.0311
	C. taiwaniana	1.17564	1.78299**	2.46865**	0.1863**	0.2043
	C. fairylakea	0.90651	1.14106	1.04925	0.2369	0.3587
	C.changjiangensis	-0.20469	-0.50692	-0.82450	0.0310	0.0271
	C. szechuanensis	-2.02240	-1.91224	-0.73556	0.0185	0.5825
FJ	*C.lingshuigensis*	0.76072	0.64156	0.05106	0.0045	0.1525
	C. hainanensis	-2.45101*	-2.53231*	-1.54449	0.0514	0.0947
	C. taiwaniana	0.99199	1.57321*	2.33880*	0.2135**	0.5622
	C. fairylakea	0.68090	1.10398	1.52961	0.0321	0.5543
	C.changjiangensis	0.84207	0.36259	-0.64761	0.0271	0.1122
	C. szechuanensis	0.17401	-0.00217	-0.43352	0.0195	0.5600

*P<0.05; **P<0.01

Supplementary Table 9. Bottleneck analysis for 18 populations of the *C. taiwaniana* complex.

Species	population	Wilcoxon test	Mode shift
C.lingshuigensis	LING1	0.219	**L**
	LING2	0.578	**L**
C. hainanensis	HAI1	0.719	**L**
	HAI2	0.078	**L**
	HAI3	0.016*	**L**
	HAI4	0.344	**L**
	HAI5	0.984	**L**
C. taiwaniana	TAI1	0.016*	**-**
C. fairylakea	FAIRY1	0.594	**L**
	FAIRY2	0.125	**-**
	FAIRY3	0.031*	**-**
	FAIRY4	0.063	**-**
	FAIRY5	0.031*	**-**
C.changjiangensis	CHA1	0.422	**L**
	CHA2	0.422	**L**
	CHA3	0.078	**L**
	CHA4	0.578	**L**
C. szechuanensis	SZE1	0.891	**L**

*P<0.05; L, normal L-shaped distribution; -, shifted mode
